# Moesin is an α_ν_β_3_ integrin-interacting protein that affects endothelial cell activation and angiogenesis through balancing c-Met and VEGFR2 activation

**DOI:** 10.1007/s00018-026-06352-5

**Published:** 2026-07-15

**Authors:** Michaela-Karina Enake, Wanjing Lai, Sotiria Tsirmoula, Κonstantinos Papadimatos, Marina Κoutsioumpa, Lamprini Skondra, Spyros Skandalis, Constantinos M. Mikelis, Lingling Shi, Evangelia Papadimitriou

**Affiliations:** 1 Laboratory of Molecular Pharmacology, Department of Pharmacy, University of Patras, Patras, GR26504 Greece; 2 Key Laboratory of CNS Regeneration (Ministry of Education), Guangdong Key Laboratory of Non-human Primate Research, GHM Institute of CNS Regeneration, Jinan University, Guangzhou, Guangdong 510632 China; 3 School of Biomedical Sciences, The University of Hong Kong, Hong Kong, China; 4 Laboratory of Biochemistry, Department of Chemistry, University of Patras, Patras, GR26504 Greece; 5 Department of Psychiatry, The First Affiliated Hospital of Jinan University, Guangzhou, Guangdong 510632 China; 6 Australis Pharmaceuticals, Inc., Simi Valley, CA USA

**Keywords:** Amoeboid cell migration, Receptor recycling, Tight junctions, VE-cadherin, ZO-1

## Abstract

Moesin (Msn) is a member of the ERM (Ezrin, Radixin, and Moesin) protein family implicated in cell-cell recognition, cell adhesion, and migration. In this work, we identified Msn as an α_ν_β_3_ integrin-interacting molecule in human endothelial cells, with a role in endothelial cell signaling and angiogenesis. Msn and its active phosphorylated form are highly expressed in endothelial cells in vitro and in vivo. Pleiotrophin and vascular endothelial growth factor A_165_ (VEGFA_165_), increase Msn phosphorylation and decrease the interaction of Msn with α_ν_β_3_ integrin. Downregulation of Msn expression in endothelial cells using siRNA reduced VEGF receptor 2 (VEGFR2) expression and activation. It also decreased permeability, collective cell migration, and tube formation in vitro, concomitant with reduced angiogenesis and blood-brain barrier permeability in *Msn* knockout mice. Concurrently, it decreased β_3_ integrin expression, increased the c-Src- and phosphoinositide 3-kinase (PI3K)-dependent cell-surface localization of nucleolin, and activated ERK1/2 kinases and cMet. Pharmacological inhibitors of c-Src, PI3K, cell-surface nucleolin, ERK1/2, and cMet, but not of VEGFR2 or α_v_β_3_ integrin, abolished siMsn-induced individual-cell motility. Taken together, these findings identify a novel binding partner of the α_v_β_3_ integrin that regulates the activation of cMet and VEGFR2 in endothelial cells, clarifying the mechanisms underlying the activation of multiple angiogenic pathways and highlighting potential new targets for improving anti-angiogenic therapies.

**Supplementary Information:**

The online version contains supplementary material available at https://doi.org/10.1007/s00018-026-06352-5.

## Introduction

Integrins mediate cell–extracellular matrix adhesion, link extracellular matrix molecules to the cytoskeleton, and initiate signaling cascades upon binding to matrix proteins or soluble ligands. Among endothelial integrins, α_ν_β_3_ has been investigated as a therapeutic target for regulating angiogenesis. This is because its expression is increased in activated and decreased in quiescent endothelial cells, playing a significant role in endothelial cell proliferation and migration during active angiogenesis [1]. Up-regulation of α_ν_β_3_ integrin during angiogenesis was initially considered to promote angiogenesis [1, 2], supported by studies in several experimental models using α_ν_β_3_ integrin antagonists [2, 3]. However, data from mice lacking the β_3_ integrin subunit show no detectable defects in normal vascular development, enhanced tumor angiogenesis [4], and upregulated vascular endothelial growth factor A (VEGFA)-induced vascular permeability [5]. Moreover, low concentrations of α_ν_β_3_ integrin antagonists enhance rather than inhibit angiogenesis, possibly by enhancing VEGF receptor 2 (VEGFR2) recycling [6]. These findings suggest that the role of α_v_β_3_ integrin in angiogenesis is multifaceted, underscoring the importance of identifying molecules involved in α_v_β_3_ integrin–mediated pathways to inform drug development.

We previously identified the α_ν_β_3_ integrin as a receptor for pleiotrophin (PTN), which is required for its enhancement of cell migration [7]. We also showed that α_ν_β_3_ integrin is essential for PTN- [8] and VEGFA_165_- [9] induced localization of nucleolin (Ncl) to the cell surface. Cell surface localization of Ncl is increased in activated endothelial cells and in several types of cancer cells, where it promotes cell motility through mechanisms that are not yet fully understood [10]. Pseudopeptides targeting cell surface Ncl markedly inhibit the migration of endothelial and cancer cells expressing α_ν_β_3_ integrin [8] and completely block PTN- [11] and VEGFA_165_- [12] induced endothelial cell chemotaxis.

The 4.1R, ezrin, radixin, and moesin (FERM) superfamily comprises proteins with a conserved structure featuring an N-terminal FERM domain that binds transmembrane proteins and lipids and a C-terminal domain that interacts with β-actin. These proteins regulate key signaling pathways involved in cell migration, survival, and differentiation. The FERM domain is present in several α_ν_β_3_ integrin–interacting proteins, including talin, kindlin, and vinculin, which control integrin-mediated adhesion and integrin activation states [13–16]. Within this family, the ERM proteins—ezrin, radixin, and moesin (Msn)—form a distinct subgroup, with Msn being the predominant member in endothelial cells [17]. Msn plays a critical role in endothelial permeability, F-actin organization, and signaling pathways that regulate cell shape, migration, and polarity [18–22].

In our search for α_ν_β_3_ integrin-interacting signaling proteins, we identified Msn and demonstrated its important role in regulating endothelial cell function and angiogenesis.

## Materials and methods

### Mice

The *Msn* knockout (KO) C57BL/6 mouse model was generated as previously described [23]. Mice were maintained under a 12-hour light/dark cycle at a controlled temperature and relative humidity, had ad libitum access to sterile food and water, bedding was changed every two weeks, and all experiments were carried out using age-matched male littermates.

### Blood-brain barrier (BBB) permeability assay

The assay was performed as previously described [24]. Briefly, postnatal day 14 (P14) male mice were anesthetized with 4% tribromoethanol (0.2 mL/10 g body weight, i.p.), and FITC-labeled dextran (4 kDa; Beyotime, #ST2930) was administered i.p. at 5 mg/mL (0.1 mL per 5 g body weight). After 1 h, mice were transcardially perfused with 40 mL phosphate-buffered saline (PBS), pH 7.4, and euthanized. Brains, excluding the cerebellum and olfactory bulbs, were homogenized in 1 mL PBS using a syringe plunger and a 40 μm cell strainer. Homogenates were centrifuged at 16,000 × g for 10 min at 4 °C, and the supernatant was mixed with an equal volume of 15% trichloroacetic acid (TCA) and centrifuged again. The resulting supernatant was neutralized with 1 N NaOH, and fluorescence was measured using a Feyond-A300 spectrophotometer (excitation/emission: 485/535 nm). Background-subtracted values were normalized to wild-type controls.

### Immunofluorescence staining in vivo

Eight-week-old wild-type and Msn KO male mice were anesthetized with 4% tribromoethanol (0.2 mL/10 g body weight, i.p.). Following transcardial perfusion with PBS, brains were carefully dissected and fixed in 4% paraformaldehyde in PBS for 48 h. Tissues were cryoprotected and sectioned coronally at 30 μm using a cryostat. Brain sections were permeabilized and blocked for 1.5 h at room temperature (RT) in PBS containing 0.15% Triton X-100, 10% saponin, and 5% donkey serum (Solarbio #SL050). Primary antibodies against Msn (1:100, Cell Signaling Technology or CST #3150), phosphorylated Msn (1:100, CST #3141), and CD31 (1:300, R&D Systems #AF3628) diluted in blocking buffer were added for 24 h at 4 °C. After washing with PBS, sections were incubated for 1.5 h at RT with Alexa-conjugated secondary antibodies (1:1000, Abcam) together with DAPI (1:3000, Merck #28718-90-3). Following PBS washes, sections were mounted and imaged using a laser scanning confocal microscope (LSM900 inverted, Zeiss) equipped with 405, 488, 561, and 640 nm lasers and 10× or 20× objectives. Images were acquired as Z-stacks with a step size of 1.2 μm and a total thickness of 12 μm. Three-dimensional reconstructions were generated using Imaris software (version 10.2, Bitplane), and vessel quantification was performed using ImageJ software [25].

### Cell culture

Human umbilical vein endothelial cells (HUVEC) were isolated and cultured as previously described [7, 9]. Cell numbers were determined by direct counting using a standard hemocytometer.

### Mass spectrometry

Three milligrams of total cell lysate protein were immunoprecipitated against α_v_β_3_ integrin, separated by SDS–PAGE, and visualized by silver staining. Selected bands were excised and subjected to in-gel tryptic digestion. Peptides were purified using C18 µZipTips and analyzed by MALDI-TOF/TOF mass spectrometry (Ultraflex, Bruker Daltonics). Protein identification was performed using the ProFound search engine with internal calibration, as previously described [8, 26].

### Biotinylation of cell membrane proteins

Cells were labeled with 0.5 mg/mL NHS-D-biotin (Vector Laboratories #QBD-10205-100) for 40 min at 4 °C, washed with ice-cold PBS, and lysed in cell lysis buffer (PBS containing 1% Triton X-100, 0.1% SDS, sodium orthovanadate, protease inhibitors, and EDTA). Total cell lysates (1 mg protein) were incubated with streptavidin magnetic beads (Roche Applied Science #11641778001) for 24 h at 4 °C, and bead-bound proteins were analyzed by Western blotting.

### Immunoprecipitation assay

Cells were washed with ice-cold PBS and lysed in cell lysis buffer. Total cell lysates (3 mg protein) were incubated overnight at 4 °C with the primary antibodies (anti-α_ν_β_3_ integrin, Merck Millipore, #MAB1976Z; anti-Msn, Santa Cruz Biotechnology Inc. or SCB, #sc-58806; anti-VEGFR2, CST, #9698) or IgG (Sigma-Aldrich, St. Louis, MO, USA #I2511 or #SAB4702080), followed by incubation with protein A/G agarose beads (Merck Millipore #IP-02 and IP-04) for 2 h. Bead-bound proteins were analyzed by Western blotting.

### Western blotting

Proteins were separated by SDS–PAGE and transferred to PVDF membranes (Macherey-Nagel #MN-741260). Membranes were blocked in Tris-buffered saline pH 7.4 − 0.05% Tween 20 (TBS-T) containing 5% nonfat dry milk or 3% bovine serum albumin (BSA) for 1 h at RT and incubated overnight at 4 °C with primary antibodies (anti-Msn, 1:500, SCB #sc-58806; anti-phospho-Msn, 1:500, SCB #sc-12895; anti-β_3_, 1:1000, SCB #sc-365679; anti-Ncl, 1:200, SCB #sc-8031; anti-Met, 1:1000, CST #8198; phospho-p44/42 MAPK and p44/p42 MAPK, 1:1000, CST #9101 and 9102; anti-VEGFR2, 1:1000, CST #9698; phospho-VEGF Receptor 2 Tyr1175, 1:500, CST #2478; ZO-1 Polyclonal Antibody, 1:5000, Proteintech #21773-1-AP; anti-beta actin, 1:1000, SCB #sc-58673) diluted in TBS-T. Following three 5-min washes with TBS-T, membranes were incubated with HRP-conjugated secondary antibodies (CST or SCB) for 2 h at RT and washed three times with TBS-T and twice with TBS. SuperSignal™ West Pico PLUS Chemiluminescent Substrate (Thermo Fisher Scientific #34580) was used to detect immunoreactive bands. Quantification was performed using ImageJ.

### Immunofluorescence

Cells were fixed with 4% formaldehyde in PBS for 10 min, permeabilized with PBS containing 0.1% Triton X-100, and washed three times with PBS. Blocking was performed in PBS containing 3% BSA and 10% fetal bovine serum (FBS) for 1 h at RT and incubated with primary antibodies (anti-β_3,_ 1:50, SCB #sc-365679; anti-Ncl−Atto 488, 1:1000, Sigma-Aldrich #N6288; anti-Ncl, 1:50, SCB #sc-8031; anti-phosphorylated Msn, 1:200, CST #3141; anti-Msn, 1:50, SCB #sc-58806; anti-VEGFR2, 1:100, CST #9698; anti-VE-cadherin, 1:200, CST #2500) diluted in PBS containing 3% BSA. Alexa-conjugated secondary antibodies (1:500, Molecular Probes) were applied, and nuclei were stained with Draq5 (3.3 µM, Biostatus Limited #DRS1000) or DAPI (1:3000, Merck #28718-90-3). Cells were mounted in Mowiol 4–88 (Sigma-Aldrich #81381) and imaged at RT using a Leica SP5 confocal microscope (40x or 65x objective). When required, mean and site-specific fluorescence intensities were quantified using the Fiji application within ImageJ.

### In situ proximity ligation assay

An in-situ proximity ligation assay (PLA) was performed to detect protein–protein interactions or protein phosphorylation. Cells were seeded in 24-well plates (4 × 10⁴ cells/well) or 12-well slides (Ibidi #81201) at 1.2 × 10⁴ cells/well. Cells were fixed with 4% formaldehyde in PBS for 10 min at RT and permeabilized with 0.05% Triton X-100 in PBS for 5 min at RT. The interaction between α_ν_β_3_ integrin and moesin was assessed using goat anti-β_3_ integrin (1:50; SCB #sc-6627) and mouse anti-Msn (1:50, SCB #sc-58806) primary antibodies. Duolink^®^ PLA probes Anti-Mouse PLUS (#DUO92001) and Anti-Goat MINUS (#DUO92006) were applied, and interactions were visualized with Duolink^®^ In Situ Detection Reagents Orange (#DUO92007). Phosphorylated cMet or VEGFR2 was detected using Naveni pY Met and Naveni pY VEGFR2 kits (Navinci, Uppsala, Sweden). Nuclei were stained with Draq5. Cells were mounted with Mowiol 4–88 and imaged at RT using a Leica SP5 confocal microscope (40x or 65x objective). Cytoplasmic size was measured using Duolink ImageTool software, and total PLA dots per cell were quantified as previously described [27].

### RNA interference

HUVEC at a confluence of 50% were transfected using annealed RNA for Msn (final concentration 50 nM; SCB #sc-35955) or for β_3_ (final concentration 33 nM; SCB #sc-35677) in medium containing FBS but no antibiotics. Lipofectamine^®^ RNAiMAX (Thermo Fisher Scientific #13778075) was the transfection reagent. Negative control siRNA (Ambion Inc. #AM4635) was used as a reference. After 48 h, cells were fixed, lysed, or serum-starved, depending on the preceding experiments.

### Transient Msn overexpression

HUVEC were transfected with pEGFP-N1 (Clontech) or GFP-tagged moesin pHJ320 (Addgene plasmid #20671, a gift from Stephen Shaw) using jetPEI-HUVEC (Polyplus Transfection #108–05 N). At 50% confluency, cells were incubated with DNA and jetPEI-HUVEC at an N/P ratio of 5 in medium containing 2% FBS for 4 h at 37 °C. Fresh medium with 30% FBS was added, and 48 h later, cells were fixed, lysed, or serum-starved, depending on the preceding experiments.

### In vitro permeability assays

For permeability assays, 2 × 10⁵ cells were seeded per well in 24-well microchemotaxis chambers (Corning #3413) with uncoated 0.4 μm polycarbonate membranes and cultured for 24 h. Medium was replaced with antibiotic-free medium, and cells were transfected with Msn siRNA as described above. After 48 h, cells were stimulated with VEGFA_165_ (10 ng/mL) in serum-free medium, and permeability was assessed at multiple time points by measuring transepithelial electrical resistance (TEER) using an EVOM2 meter with STX2 chopstick electrodes, according to the manufacturer’s instructions.

### Scratch assay

Cells were seeded in 24-well plates at 2 × 10⁴ cells/well and incubated for 24 h before Msn knockdown using siRNA, as described above. Forty-eight hours post-transfection, a 100–1000 µL pipette tip was used to create a scratch wound. Wound closure was monitored and photographed with an OPTIKA CB3 camera at 0 h and at subsequent time points until complete closure. Cell migration was quantified by measuring the remaining wound area in ImageJ and expressing it as a percentage of the initial wound area at 0 h.

### Transwell assay

Individual cell motility assays were conducted using 24-well microchemotaxis chambers (SPL Life Sciences #35224) with uncoated 8 μm polycarbonate membranes. The lower chamber contained 0.6 mL serum-free medium with 0.25% BSA. Cells were resuspended at 1 × 10⁵ cells/0.1 mL in the same medium, added to the upper chamber, and incubated for 4 h at 37 °C. Chemotactic agents were added to the lower chamber only, while pharmacological inhibitors, when used, were added to both chambers. Filters were fixed with 4% formaldehyde in PBS for 10 min and stained with 0.1% crystal violet. Migrated cells were quantified across the entire filter using a light microscope [7, 9].

### Matrigel tube formation assay in vitro

Matrigel™ (BD Matrigel™ Basement Membrane Matrix, Corning, NY 14831 USA #354234) was added (0.05 ml) to each well of 96-well plates at 4°C and left to solidify at 37 °C for 1 h. 2 × 10^4^ HUVEC resuspended in 0.1 ml of medium were added in each well and incubated at 37 °C for 6 h. The tubes formed were photographed under an inverted Olympus IMT-2 microscope (Olympus Corporation, Japan) using a C-B3 camera (Optica Microscopy, Italy). The total length of the formed tubes, the total length of the segments, and the total number of junctions were quantified in the total area of the wells using the Angiogenesis Analyzer for ImageJ.

### Statistical analysis

Statistical comparisons between two or among three or more groups were performed using an unpaired Student’s T-test or one-way ANOVA, respectively.

## Results

### Msn interacts with α_ν_β_3_ integrin

**Fig. 1:**
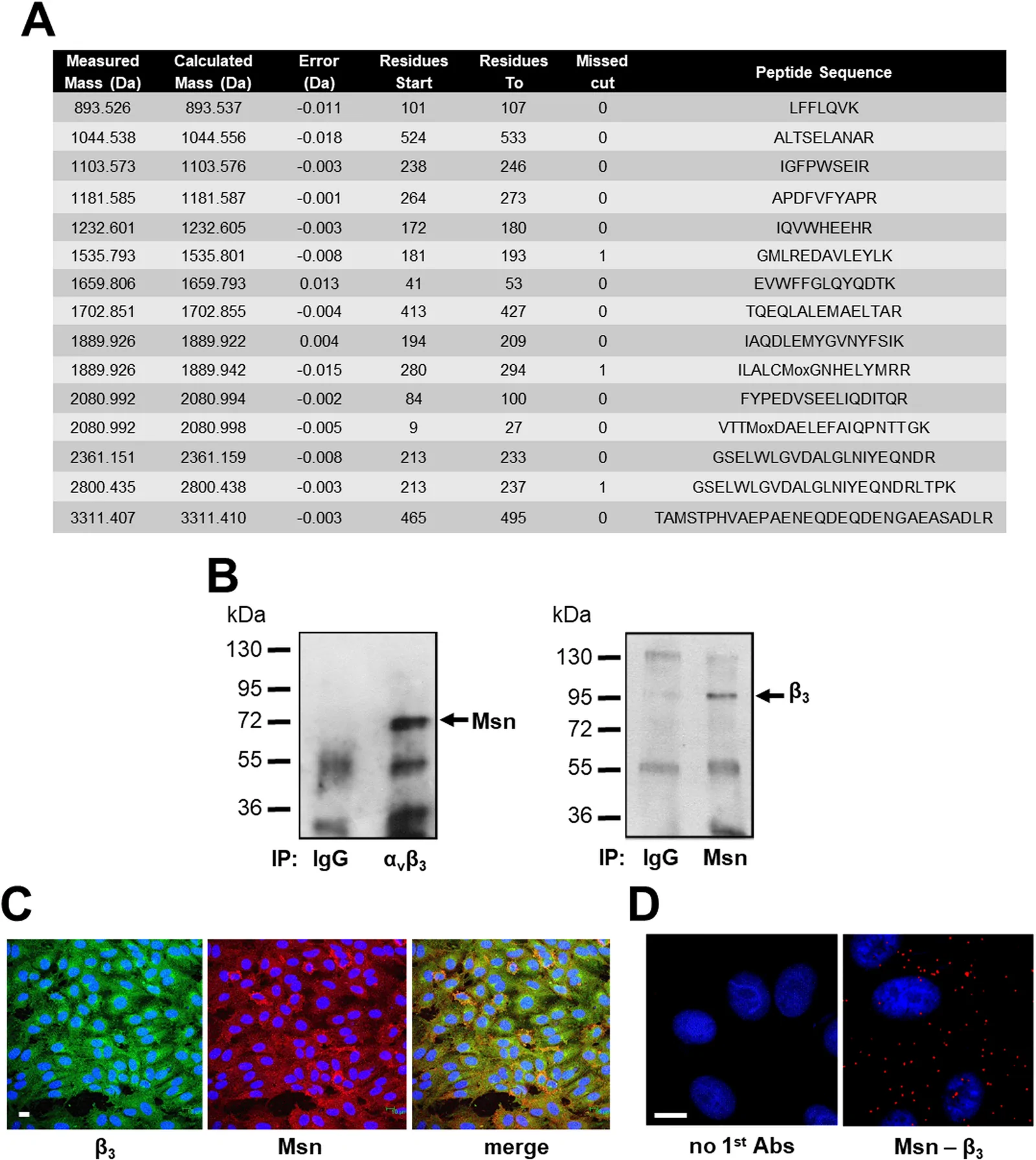
Msn interacts with α_ν_β_3_integrin in HUVEC. **A**Msn was identified by peptide mass fingerprint analysis following immunoprecipitation with an anti-α_v_β_3_ integrin antibody. The table shows 18 tryptic peptides covering 42% of the human Msn sequence. **B**HUVEC lysates were immunoprecipitated with antibodies against α_ν_β_3_ integrin or Msn, followed by Western blotting to detect Msn or β_3_ integrin, respectively. IgG served as a negative control. Blots represent four independent experiments. **C **Immunofluorescence staining of HUVEC for β_3_ integrin (green), Msn (red), and nuclei (blue). Representative images from three independent experiments are shown. **D** In situ PLA demonstrating Msn-β_3_ integrin complexes (red dots) in HUVEC. Nuclei are stained in blue. Representative images from three independent experiments. Scale bars in (**C**) and (**D**) = 10 μm

To identify α_ν_β_3_ integrin-interacting proteins that may elucidate how α_ν_β_3_ integrin regulates endothelial cell migration, total protein cell lysates were immunoprecipitated with an antibody for α_ν_β_3_ integrin and analyzed by MALDI-TOF, as described in Methods. Among the proteins co-immunoprecipitated with α_ν_β_3_ integrin, a ~ 70 kDa protein band was identified that contained peptides identical to human Msn (18 matched peptides in total of 54, minimum sequence coverage 42%) (Fig. 1A). This interaction was verified by immunoprecipitating HUVEC lysates with antibodies against α_ν_β_3_ integrin or Msn and performing Western blot analyses of the immunoprecipitates for Msn or β_3_ integrin respectively (Fig. 1B). Co-localization of β_3_ integrin and Msn was also observed on the cell membrane by immunofluorescence assays (Fig. 1C). At the same time, PLA demonstrated formation of β_3_ integrin-Msn complexes (Fig. 1D).

### PTN and VEGFA increase Msn phosphorylation and decrease Msn-β_3_integrin interaction

**Fig. 2:**
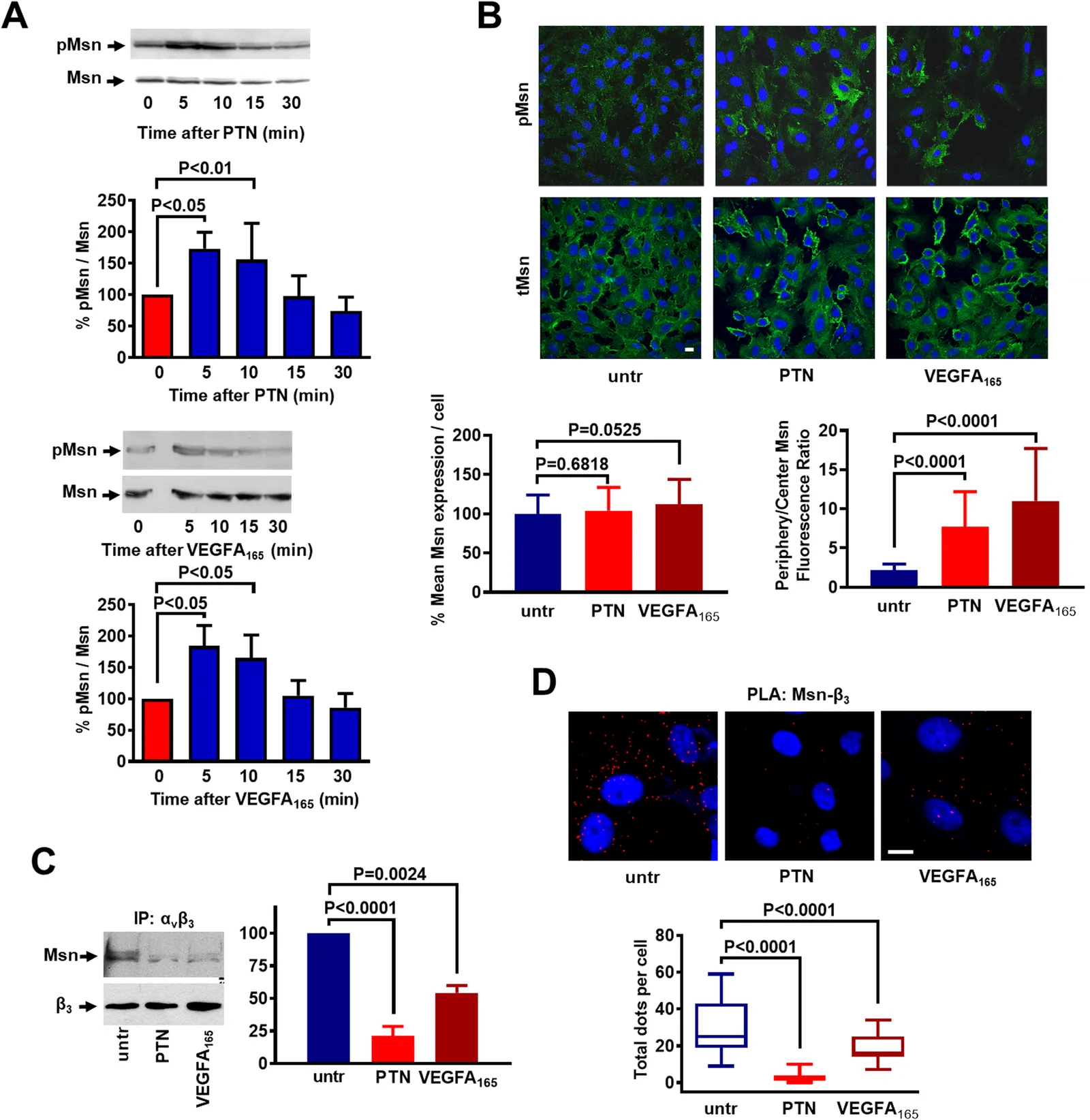
PTN and VEGFA enhance Msn phosphorylation and reduce Msn-β_3_integrin interaction. **A**Western blot of HUVEC lysates showing PTN (100 ng/mL) and VEGFA_165_ (10 ng/mL) effects on Msn Thr558 phosphorylation. pMsn/tMsn ratios are expressed relative to untreated cells (mean ± SD, *n* = 3). **Β** Immunofluorescence of serum-starved HUVEC for pMsn or tMsn (green) and nuclei (blue) after 10 min stimulation with PTN or VEGFA_165_. Scale bar = 10 μm. Mean Msn fluorescence and Periphery/Center Msn Fluorescence Ratio per cell were quantified and expressed as mean ± SD (*n* = 3). **C** HUVEC lysates post-stimulation with PTN or VEGFA_165_ were immunoprecipitated using an anti-α_ν_β_3_ integrin antibody, followed by Western blotting for Msn and β_3_ integrin. Results are expressed as mean ± SD (*n* = 3) of the percent Msn/β_3_ ratio relative to untreated cells (untr, set at 100%). **D** In situ PLA detecting Msn-β_3_ integrin complexes (red dots) in the absence or presence of PTN or VEGFA_165_ for 10 min. Nuclei were stained with Draq5 (blue). Scale bar = 10 μm. Images represent three independent experiments, and box plots derive from > 20 fields (~ 4 cells/field), each sample in duplicate

Msn is highly expressed in endothelial cells, as shown in our data (Fig. 1) and supported by previous literature [17] and the Human Protein Atlas. Single-cell data from the latter show that Msn is expressed in all types of endothelial cells, arterial, venous, and capillaries, and depending on the tissue, its expression is high in capillaries, e.g., in the lung, breast and prostate, or is higher in larger vessels, e.g., in the brain (Single cell type - MSN - The Human Protein Atlas and thereafter examples in Supplementary Fig. S1) [28]. The latter was also observed in our sections of the mouse cortex, in which Msn and its activated, phosphorylated form at Thr558 (pMsn) are localized on venules/arterioles (10–20 μm in diameter) and less on capillaries (< 5 μm) (Supplementary Fig. S2A). Co-localization of Msn and pMsn with CD31-positive cells was also observed in the cortex of P7 mice (Supplementary Fig. S2B). At this developmental stage, vascular development is highly active, with a dense, immature capillary plexus undergoing rapid angiogenesis [29], suggesting that both Msn and its phosphorylated form play important roles in angiogenesis. Angiogenic growth factors, such as PTN and VEGFA_165_, increased Msn Thr558 phosphorylation, without affecting the total amounts of Msn (Fig. 2A and B). An interesting observation was that treatment with PTN and VEGFA_165_ led to increased periphery-specific Msn localization, which was mainly observed in cells forming blebs (Fig. 2B). Although Msn has been previously linked to membrane blebbing related to T lymphocyte migration [30], functional significance and underlying mechanisms of this Msn localization in endothelial cells require further targeted investigation and are beyond the scope of the present study. PTN and VEGFA_165_ also decreased the interaction between Msn and β_3_ integrin in HUVEC. The latter was investigated by stimulating serum-starved HUVEC with PTN or VEGFA_165_ for 10 min at concentrations that increase cell migration. Total cell lysates were immunoprecipitated using an antibody against α_ν_β_3_ integrin and subjected to Western blot analysis for Msn. As shown in Fig. 2C, both PTN and VEGFA_165_ significantly decreased the co-immunoprecipitation of Msn with α_ν_β_3_ integrin. They also reduced Msn-β_3_ integrin interaction, as assessed by PLA assays (Fig. 2D).

### Msn downregulation significantly affects the motility, tube formation, and permeability of endothelial cells in vitro

**Fig. 3:**
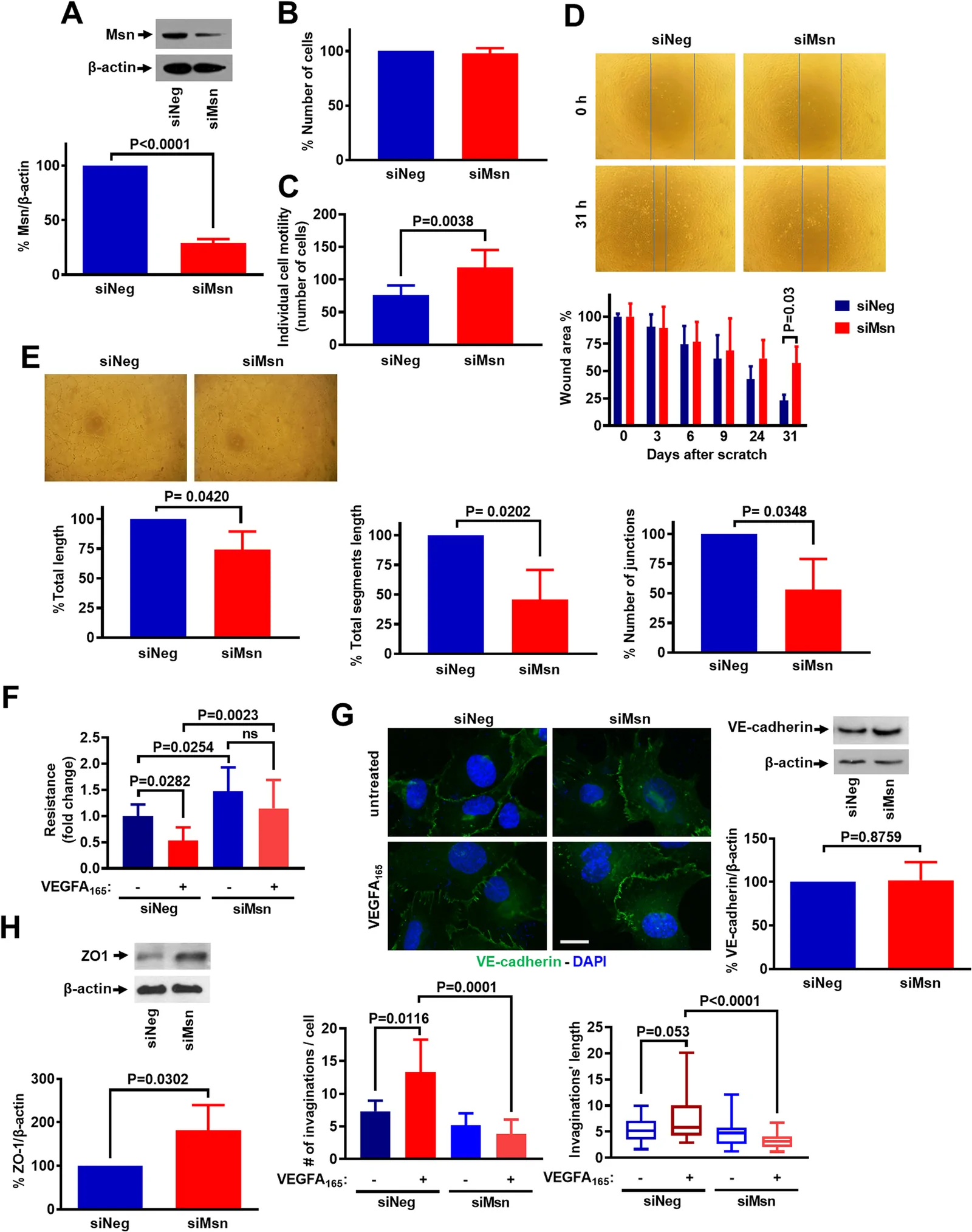
Msn downregulation alters endothelial cell motility, permeability, and tube formation in vitro. **A** Representative Western blot of total HUVEC lysates following Msn downregulation. Beta (β)-actin served as the loading control. Results are expressed as mean ± SD (*n* = 3) relative to siNeg cells (set to 100%). **B** Cell numbers were determined 48 h after siRNA transfection using a hemocytometer (percent mean ± SD relative to siNeg cells, set to 100%, *n* = 3). **C** Individual cell motility was assessed using a transwell migration assay (mean ± SD, *n* = 7). **D** Representative images of wound-healing assays at 0 h and 31 h. Wound areas were quantified using ImageJ and expressed as a percentage of the initial wound area. Data represent mean ± SD (*n* = 3). **E** Representative photos and quantification of the tube network formed by HUVEC on Matrigel. Results are expressed as mean ± SD of the percent tube length, segment length, and number of junctions compared to siNeg cells (set as default 100%). **F** TEER measurements were used to evaluate paracellular permeability. Data are shown as mean ± SD (*n* = 4) of fold changes relative to untreated siNeg cells. siNeg, cells transfected with a negative control siRNA; siMsn, cells transfected with siRNA for Msn. **G** Representative immunofluorescence images for VE-cadherin following 10 min stimulation of HUVEC with VEGFA_165_ (10 ng/ml). The data were obtained by quantifying the number and length (arbitrary units) of the invaginations using ImageJ. Measurements were performed in two different areas, each containing 3–5 cells, and are expressed as mean ± SD (*n* = 3). Representative Western blot of total HUVEC lysates for VE-cadherin following Msn downregulation. Results are expressed as mean ± SD (*n* = 3) relative to siNeg cells (set to 100%). **H** Representative Western blot of total HUVEC lysates for ZO-1 following Msn downregulation. Results are expressed as mean ± SD (*n* = 3) relative to siNeg cells (set to 100%)

To investigate whether Msn affects endothelial cell functions related to angiogenesis, we downregulated its expression using siRNA and investigated its effects on various cellular functions. Msn expression was significantly downregulated (Fig. 3A) without affecting HUVEC proliferation (Fig. 3B). HUVEC with decreased Msn expression migrated to a larger extent in the transwell assay (Fig. 3C) but to a lesser extent in the scratch assay (Fig. 3D), suggesting that Msn differentially affects individual and collective migration. The ability of HUVEC to form tubes in the Matrigel assay was significantly impaired when the expression of Msn was downregulated, as shown by the decreased total length, total segment length and number of junctions (Fig. 3E). Decreased Msn expression led to increased endothelial cells’ resistance as estimated by the TEER measurement (reduced permeability) and abrogated the VEGFA_165_-induced permeability (Fig. 3F). The maximal effect of VEGFA_165_ on siNeg HUVEC permeability was observed 75 min after stimulation of cells with VEGFA_165_ (Supplementary Fig. S3), and this is the time point when all measurements at Fig. 3F were made. Along the same line, in siNeg-treated cells, 10 min stimulation with VEGFA_165_ resulted in VE-cadherin remodeling appearing as invaginations, which did not form in siMsn-treated HUVEC. Both the number and the length of the invaginations were increased by VEGFA_165_ in siNeg-treated cells (Fig. 3G). Although it is not clear how downregulation of Msn expression affects baseline TEER, we found that ZO-1 levels are increased in siMsn-treated compared with siNeg HUVEC (Fig. 3H), supporting this observation. Total VE-cadherin levels were not affected by siMsn in HUVEC (Fig. 3G).

### Decreased angiogenesis and BBB permeability in the Msn knockout mouse

**Fig. 4:**
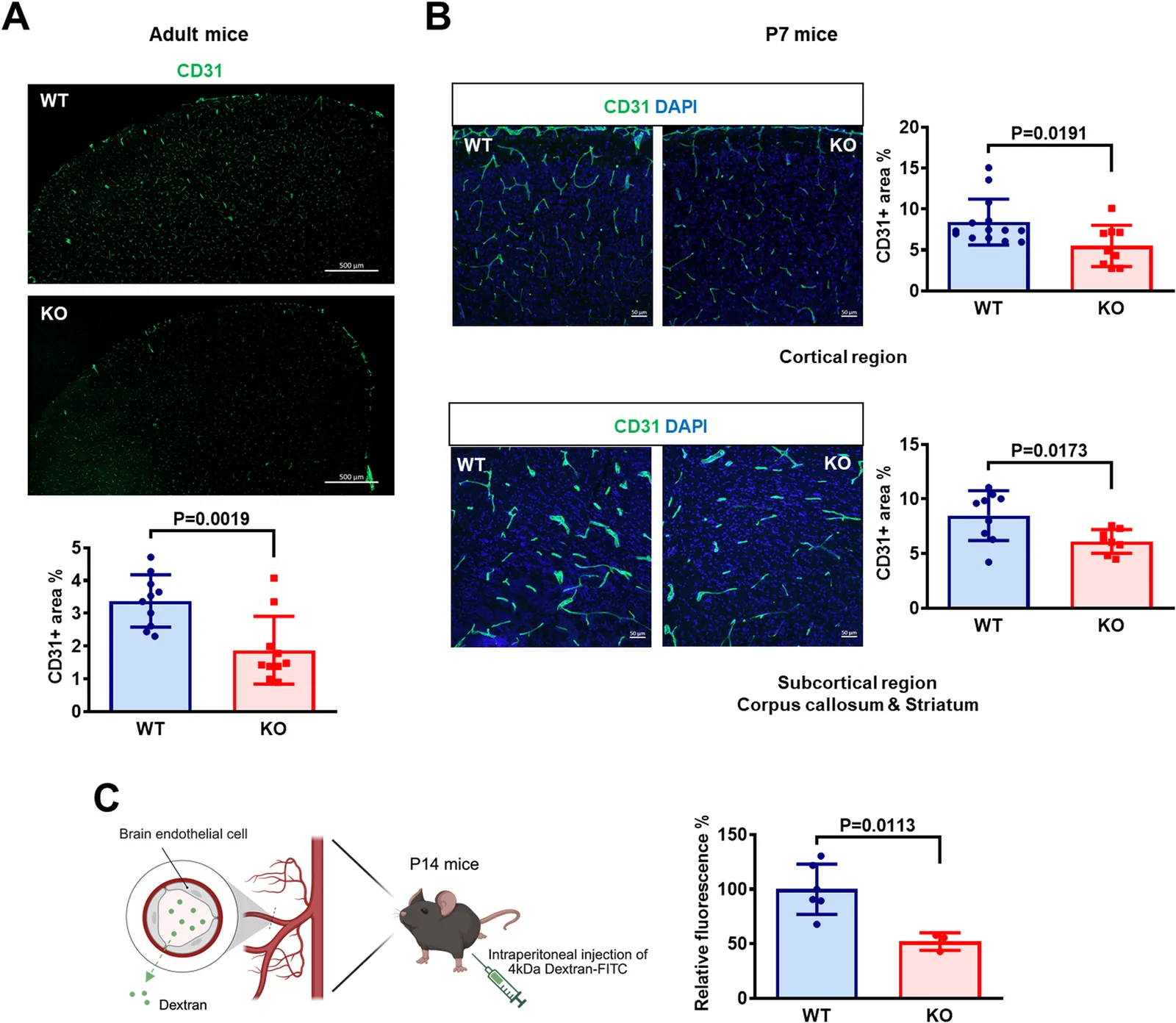
Angiogenesis and BBB permeability are decreased in Msn KO mice. **A** Decreased brain endothelial cell coverage in Msn KO adult male mice. Confocal-Tiles, 10X, scale bar = 500 μm. **B** Decreased brain endothelial cell coverage in Msn KO P7 mice. Confocal-Tiles, 10X, scale bar = 50 μm. At least 3 slides per animal and 3 animals per group were analyzed. Results are expressed as mean ± SD of the percent area covered by CD31 + cells. **C** Decreased BBB permeability in Msn KO P14 mice. The picture on the left was generated by Biorender (https://www.biorender.com/) and summarizes the experimental approach. Fluorescence intensity was normalized to the mean WT level. n(WT) = 6, n(KO) = 3. Results are expressed as mean ± SD of the percent relative fluorescence

To assess whether Msn expression influences angiogenesis in vivo, we analyzed cryosections of the brain cortex from adult male Msn knockout mice and their corresponding wild-type controls, stained with an antibody against CD31, an endothelial cell marker. As shown in Fig. 4A, there is a significant decrease in brain endothelial cells in the cortex of Msn KO compared with the wild-type mice. We did the same in cryosections of the brain cortex and the subcortical region (corpus callosum & striatum) from wild-type and knockout mice at the developmental stage P7, when the vasculature is highly dynamic, characterized by a dense, immature capillary plexus that is actively undergoing angiogenesis [29]. CD31 coverage was found significantly reduced in Msn KO mice in both the cortical and subcortical regions (Fig. 4B), supporting the notion that Msn positively regulates angiogenesis.

The BBB permeability was studied in P14 Msn KO male mice and was significantly reduced compared with that in the corresponding wild-type mice (Fig. 4C).

### Downregulation of Msn expression decreases β_3_ integrin protein levels and activates cMet

**Fig. 5:**
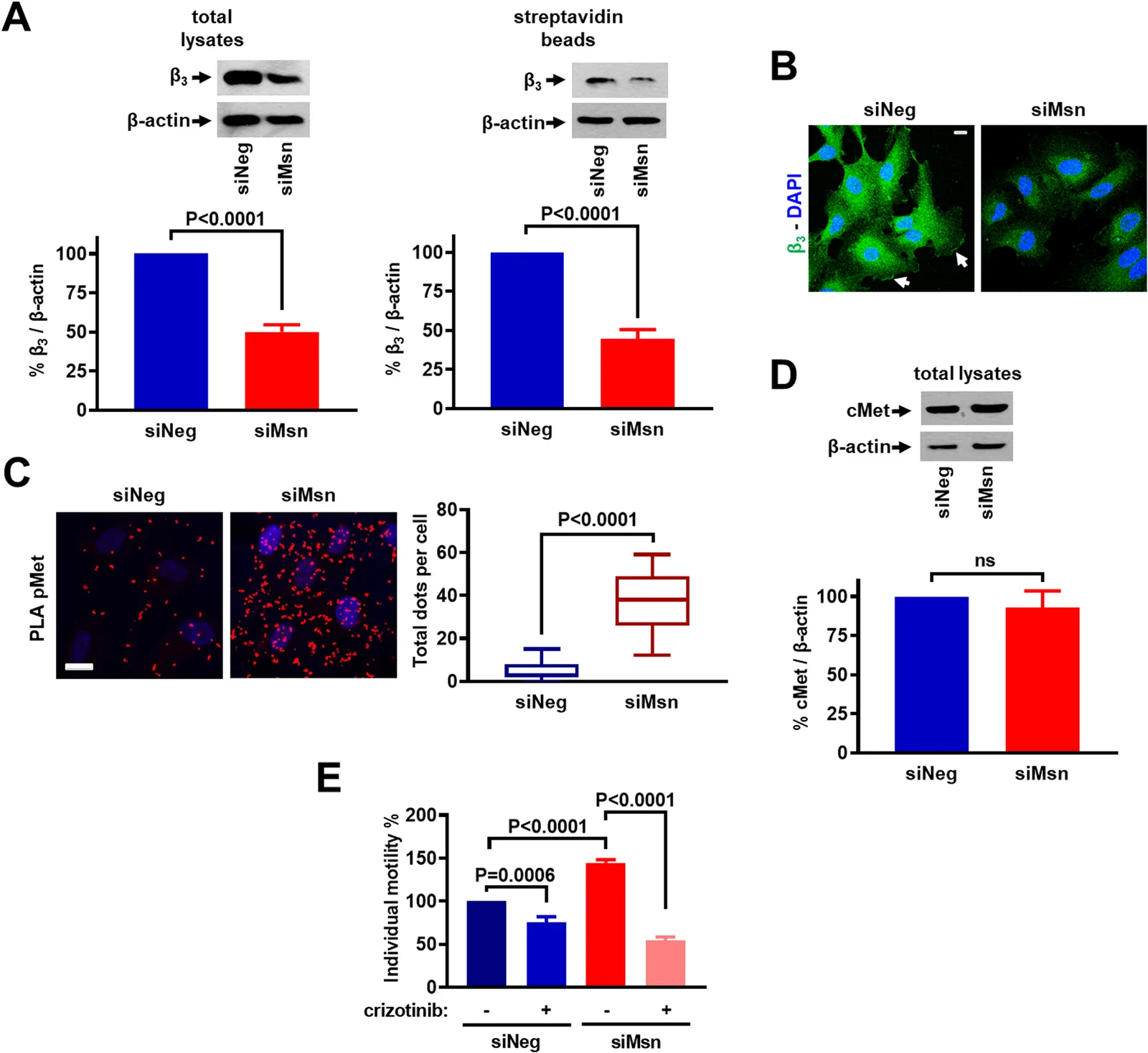
Downregulation of Msn reduces β_3_integrin protein levels and activates cMet. **A** Total HUVEC lysates or membrane protein fractions obtained by cell-surface biotinylation and streptavidin pull-down were analyzed by Western blot for β_3_ integrin, with β-actin as the loading control. Results are expressed as mean ± SD (*n* = 3) relative to siNeg cells (set to 100%). **B** Immunofluorescence localization of β_3_ integrin (green) in HUVEC. White arrows indicate cell surface–localized β_3_ integrin. Nuclei were stained with Draq5 (blue). Images represent three independent experiments. Scale bar = 10 μm. **C** In situ detection of cMet tyrosine phosphorylation in HUVEC treated for 48 h with siNeg or siMsn. Red dots indicate phosphorylated cMet sites; nuclei are shown in blue (Draq5). Scale bar = 10 μm. Images represent three independent experiments, and box plots were generated from > 20 image fields (4–8 cells per field per condition), with samples analyzed at least in duplicate. **D** Total HUVEC lysates were analyzed by Western blot for cMet and β-actin (loading control). Results are expressed as mean ± SD (*n* = 3) relative to siNeg cells (set to 100%). **E** Following Msn downregulation, HUVEC were treated with crizotinib (1 µΜ), and individual cell motility was assessed using a transwell migration assay. Results are expressed as mean ± SD (*n* = 3) and presented as percentages relative to untreated siNeg cells (set to 100%). siNeg, cells transfected with a negative control siRNA; siMsn, cells transfected with siRNA for Msn

Based on our observation that PTN or VEGFA_165_ decreases Msn interaction with α_ν_β_3_ integrin, we investigated whether Msn downregulation by siRNA affects β_3_ integrin protein levels. By Western blot analysis, we studied both the total and the membrane-bound levels of β_3_ integrin and found that they were both significantly decreased in siMsn-treated HUVEC compared to the corresponding siNeg-treated cells (Fig. 5A). The decreased β_3_ integrin levels were also evident by immunofluorescence (Fig. 5B). Treatment of cells with chloroquine, which impairs lysosomal function, reversed this effect of Msn downregulation (Supplementary Fig. S4), and overexpression of Msn led to increased β_3_ integrin protein levels (Supplementary Fig. S5).

In line with our previous data showing that decreased β_3_ integrin levels activate cMet in endothelial cells [25], downregulation of Msn expression by siRNA led to a significant activation of cMet (Fig. 5C), without affecting its levels (Fig. 5D). The cMet inhibitor crizotinib abolished the enhanced motility due to decreased Msn expression (Fig. 5E), suggesting that cMet is responsible for this effect. The crizotinib concentration used did not affect cell viability [25]. Notably, PTN or VEGFA_165_ did not affect the enhanced motility of cells following the downregulation of Msn expression (Supplementary Fig. S6).

### Downregulation of Msn expression decreases VEGFR2 protein levels

**Fig. 6:**
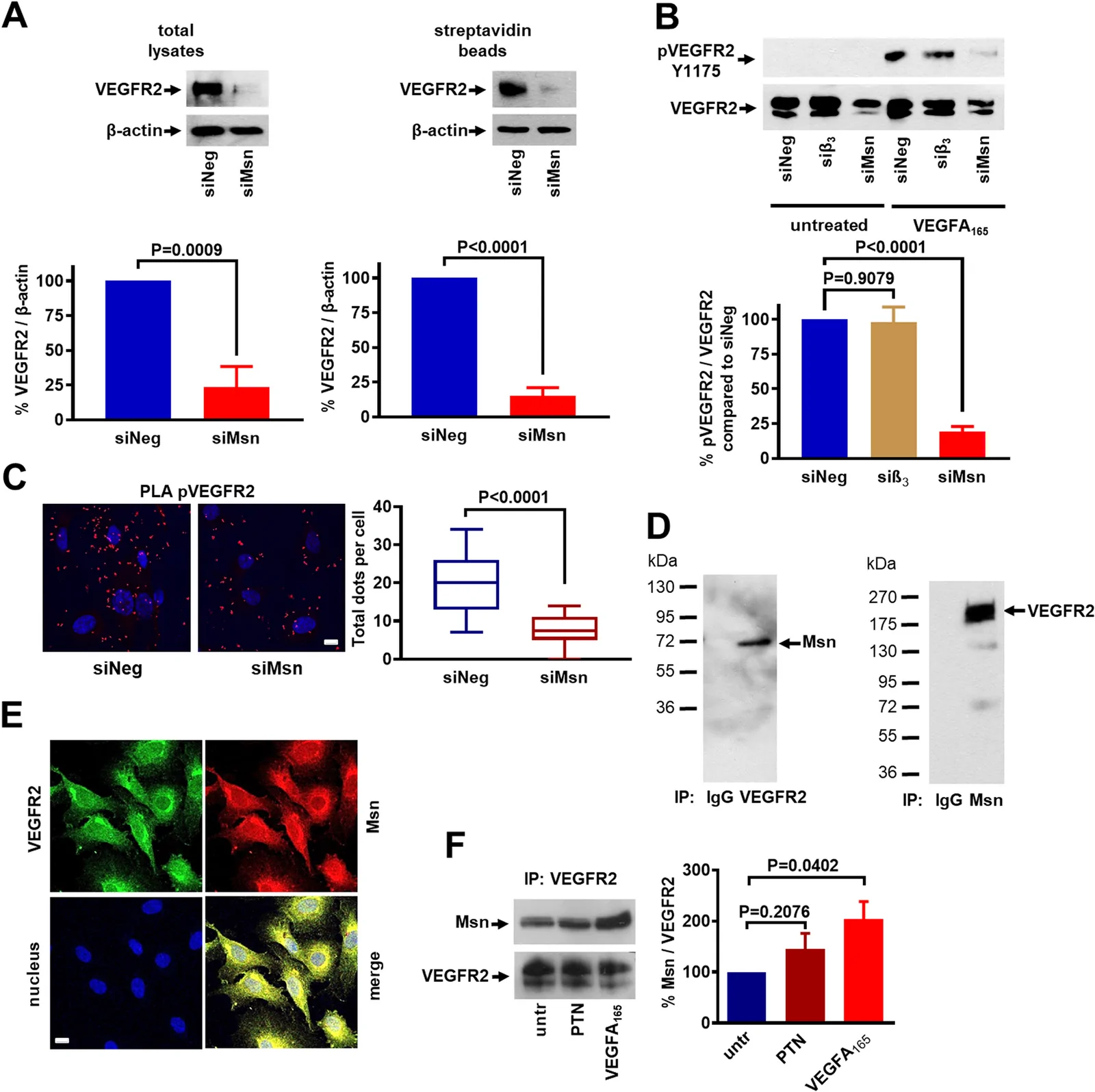
Downregulation of Msn reduces VEGFR2 protein levels and impairs VEGFR2 activation. **A** Total HUVEC lysates or membrane protein fractions obtained by cell-surface biotinylation and streptavidin pull-down were analyzed by Western blot for VEGFR2, with β-actin as the loading control. Results are expressed as mean ± SD (*n* = 3) relative to siNeg cells (set to 100%). **B** Serum-starved HUVEC were stimulated with VEGFA_165_ (10 ng/mL) for 10 min, followed by Western blot analysis of total lysates for VEGFR2 phosphorylated at Tyr1175 (pVEGFR2 Y1175) and total VEGFR2. Results are expressed as mean ± SD (*n* = 3) relative to VEGFA_165_-treated siNeg cells (set to 100%). **C** In situ detection of VEGFR2 tyrosine phosphorylation in HUVEC treated for 48 h with siNeg or siMsn. Red dots indicate phosphorylated VEGFR2 sites; nuclei are stained with Draq5 (blue). Scale bar = 10 μm. Images represent three independent experiments, and box plots were generated from > 20 image fields (4–8 cells per field per condition), with samples analyzed at least in duplicate. **D** HUVEC lysates immunoprecipitated with antibodies against VEGFR2 or Msn were analyzed by Western blot for Msn or VEGFR2, respectively. IgG served as a negative control. Images represent three independent experiments. **E** Representative immunofluorescence images showing VEGFR2 (green), Msn (red), and nuclei (blue). Yellow indicates co-localization of VEGFR2 and Msn. Images are from three independent experiments. Scale bar = 10 μm. **F** HUVEC lysates following stimulation with PTN (100 ng/mL) or VEGFA_165_ (10 ng/mL) were immunoprecipitated with an anti-VEGFR2 antibody and analyzed by Western blot for Msn and VEGFR2. Results are expressed as mean ± SD (*n* = 3) of the percent Msn/VEGFR2 ratio relative to untreated cells (set to 100%). siNeg, cells transfected with a negative control siRNA; siMsn, cells transfected with siRNA for Msn

Since β_3_ integrin levels in endothelial cells have been shown to regulate VEGFA signaling through VEGFR2 [4], we investigated whether Msn downregulation by siRNA affects the protein levels or the signaling downstream of VEGFR2. By Western blot analysis, we studied both the total and the membrane-bound levels of VEGFR2 and found them significantly decreased in siMsn-treated HUVEC compared to the corresponding siNeg-treated cells (Fig. 6A). Similarly to β_3_ integrin, this effect was reversed by chloroquine (Supplementary Fig. S4), and overexpression of Msn led to increased VEGFR2 protein levels (Supplementary Fig. S5). Because of decreased VEGFR2 protein levels, VEGFR2 phosphorylation at Tyr1175 following HUVEC stimulation with VEGFA_165_ was significantly impaired in siMsn-treated HUVEC (Fig. 6B). Similarly, the global tyrosine phosphorylation of VEGFR2 decreased in siMsn-treated HUVEC compared to the corresponding siNeg-treated cells (Fig. 6C). Downregulation of β_3_ integrin by siRNA did not affect either the protein levels or the phosphorylation of VEGFR2 (Fig. 6B), suggesting that this effect is independent of the β_3_ integrin levels. In line with these data, the specific VEGFR2 tyrosine kinase inhibitor SU1498 (Supplementary Fig. S7A) and inhibitors of α_ν_β_3_ integrin (Supplementary Fig. S7B) inhibited cell migration on their own but not the siMsn-induced individual endothelial cell motility. Interestingly, Msn was found to co-immunoprecipitate (Fig. 6D) and co-localize with VEGFR2 in endothelial cells (Fig. 6E), and their interaction was increased by VEGFA_165_ (Fig. 6F).

### Downregulation of Msn expression enhances the cell surface translocation of Ncl

**Fig. 7:**
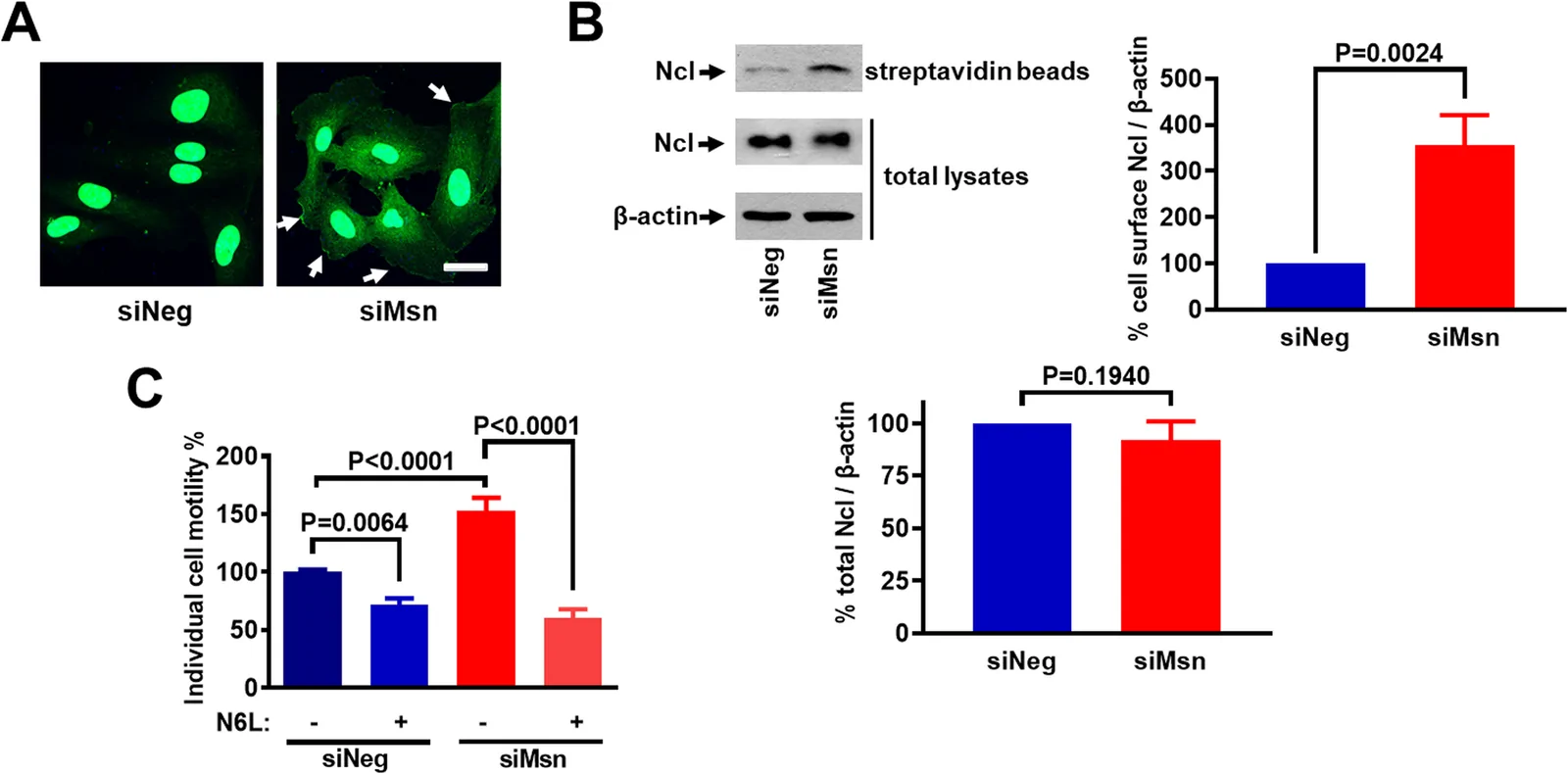
Downregulation of Msn enhances cell surface translocation of Ncl. **A** Immunofluorescence localization of Ncl (green) in HUVEC. White arrows indicate increased cell surface localized Ncl in cells with reduced Msn expression. Nuclei were stained with Draq5 (blue). Images represent three independent experiments. Scale bar = 10 μm. **B** Total HUVEC lysates or membrane protein fractions obtained by cell-surface biotinylation and streptavidin pull-down were analyzed by Western blot for Ncl, with β-actin as the loading control. Results are expressed as mean ± SD (*n* = 3) relative to siNeg cells (set to 100%). **C** Following Msn downregulation, HUVEC were treated with N6L (10 µg/mL), and cell motility was assessed using a transwell migration assay. Results are expressed as mean ± SD (*n* = 3) as percentages relative to untreated siNeg cells (set to 100%)

Both PTN and VEGFA_165_ have been shown to stimulate translocation of Ncl, increasing its cell-surface expression [8, 9] in an α_ν_β_3_ integrin-dependent, VEGFR2-independent manner [9]. Cell-surface Ncl is essential for the stimulatory actions of both growth factors [11, 12]. In the present work, we examined whether Ncl is affected and plays a role in the enhanced individual motility following Msn downregulation. Down-regulation of Msn expression by siRNA in HUVEC resulted in increased Ncl levels on the cell membrane, as evidenced by both immunofluorescence (Fig. 7A) and Western blot analysis of biotinylated proteins (Fig. 7B), without affecting the total protein levels of Ncl (Fig. 7B). The pseudopeptide N6L that binds cell surface Ncl and inhibits cell migration stimulated by PTN or VEGFA_165_ [8, 10, 12] abolished the stimulatory effect of the downregulation of Msn expression (Fig. 7C) on individual endothelial cell motility, suggesting that cell surface Ncl is important for this effect.

### Signaling downstream of Msn downregulation that leads to Ncl translocation to the cell surface and cMet activation

**Fig. 8:**
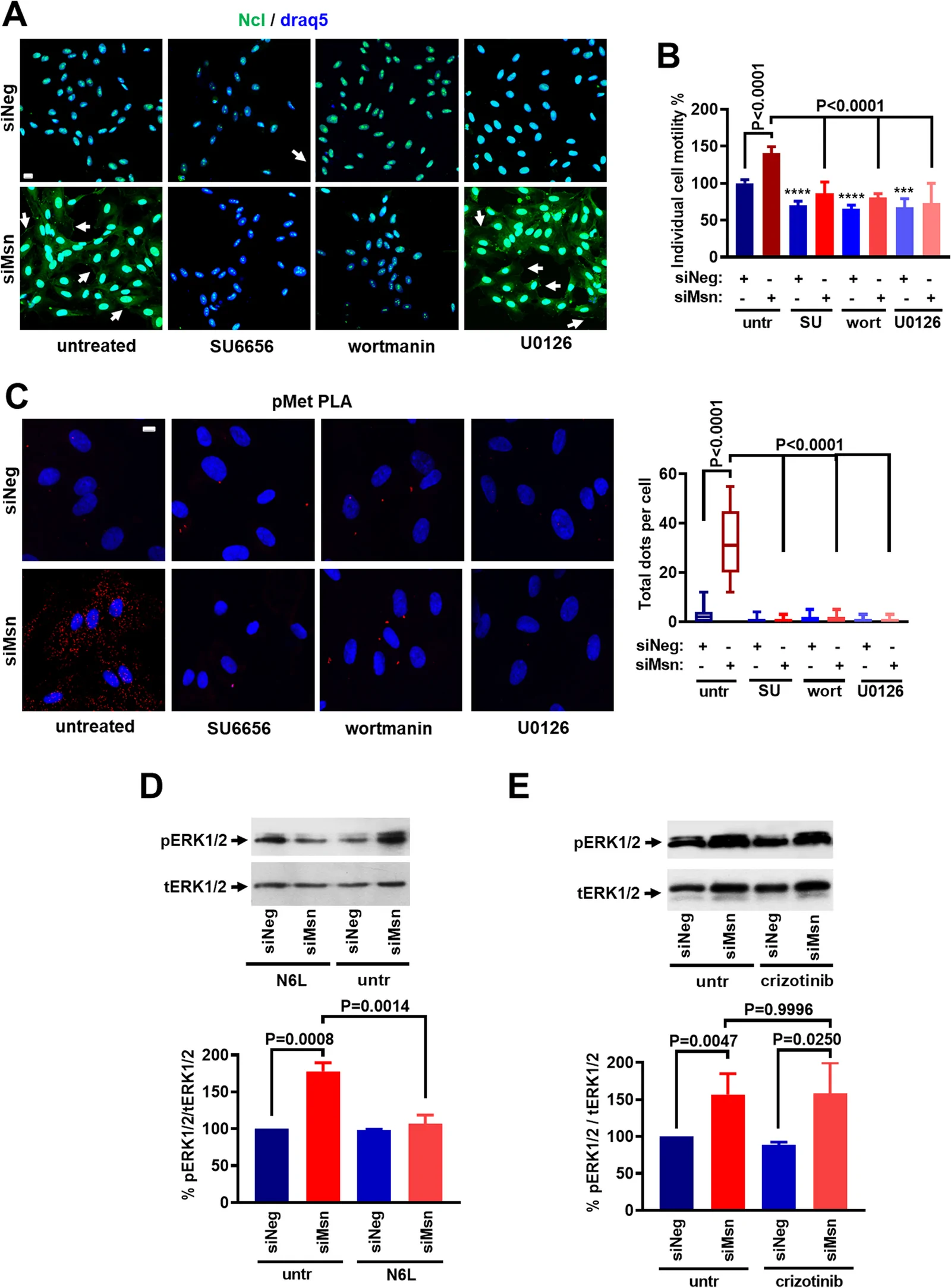
Signaling pathways downstream of Msn downregulation. **A** Immunofluorescence localization of Ncl (green) in HUVEC following Msn knockdown by siRNA. Cells were treated for 24 h with SU6656 (10 µM), wortmannin (100 nM), or U0126 (20 nM), then fixed and stained for Ncl (green) and nuclear DNA (blue). Images represent three independent experiments. Scale bar = 20 μm. **B** Following Msn downregulation, HUVEC were treated with the indicated inhibitors, and individual cell motility was assessed using a transwell migration assay. Results are expressed as mean ± SD (*n* ≥ 3) percentages relative to untreated siNeg cells (set to 100%). **C** In situ PLA detecting cMet tyrosine phosphorylation in HUVEC treated for 48 h with siNeg or siMsn in the presence or absence of the indicated inhibitors. Red dots represent phosphorylated cMet sites; nuclei are stained with Draq5 (blue). Scale bar = 10 μm. Images representat three independent experiments, and box plots were generated from > 20 image fields (4–8 cells per field per condition), with samples analyzed at least in duplicate. **D**,** E** Following Msn downregulation, HUVEC were treated for 24 h with N6L (10 µg/mL) or crizotinib (1 µΜ). Total cell lysates were analyzed by Western blot for phosphorylated ERK1/2 (pERK1/2) and total ERK1/2 (tERK1/2). Data are expressed as mean ± SD (*n* = 3) of the percent pERK1/2/tERK1/2 ratio relative to untreated siNeg cells (set to 100%)

We have previously shown that downstream of α_ν_β_3_ integrin, PTN and VEGFA_165_ enhance Ncl translocation to the cell membrane through c-Src and PI3K, but not MAP kinases [8, 9]. We therefore investigated whether pharmacological inhibitors of these kinases affect Ncl translocation to the cell surface in the context of Msn downregulation. As shown in Fig. 8A, SU6656, which selectively inhibits c-Src, and the PI3K inhibitor wortmannin abolished the cell surface translocation of Ncl, whereas the MEK1 inhibitor U0126 had no effect. However, all three inhibitors abolished the enhanced motility caused by Msn downregulation (Fig. 8B). We therefore examined their influence on cMet activation. We found that all three abolished the stimulatory effect of Msn downregulation (Fig. 8C), suggesting that they are all upstream of cMet activation. These data suggest that ERK1/2 kinases are activated downstream of the translocation of Ncl to the cell membrane, and indeed, N6L abolishes the siMsn-induced ERK1/2 activation (Fig. 8D). Crizotinib did not affect ERK1/2 activation due to Msn downregulation (Fig. 8E), further suggesting that cMet is downstream of ERK1/2 activation in this pathway.

## Discussion

To clarify the α_ν_β_3_ integrin-related signaling pathway in endothelial cells, we searched for proteins that interact with α_ν_β_3_ integrin and identified Msn as one of them. The interaction between Msn and β_3_ integrin occurs at the cell membrane and is inhibited by PTN or VEGFA_165_, suggesting that this interaction is transient, affected by growth factor stimulation, and may regulate endothelial cell functions. We therefore downregulated Msn expression in endothelial cells and observed significantly decreased β_3_ integrin levels and enhanced cMet activation, consistent with our previous findings [25] and those of others [31], which have linked decreased β_3_ integrin levels to elevated cMet activity. The activation of cMet aligns with the membrane blebbing and the enhanced individual endothelial cell motility observed in the absence of any chemotactic agent in the transwell assay, according to previous studies showing membrane blebbing due to cMet activation in tumor cells [32–34], which leads to enhanced amoeboid cell motility [35]. Endothelial cells have been shown in vitro and in vivo to migrate in both mesenchymal and amoeboid modes, with the latter being independent of the VEGFA canonical signaling [36]. We have previously demonstrated that in parallel to VEGFR2, VEGFA activates cMet through receptor protein tyrosine phosphatase zeta 1 (PTPRZ1) [25]. This study demonstrates that endothelial cell individual motility, resulting from downregulated Msn expression, does not respond to VEGFA stimulation in the transwell motility assay, and VEGFR2 tyrosine kinase inhibition does not affect the siMsn-induced motility. These data suggest that VEGFA, through cMet, activates amoeboid endothelial cell motility, supporting our previous data showing that crizotinib abolished this effect of VEGFA_165_ in HUVEC [25]. However, this pathway is insufficient in vivo to sustain angiogenesis in the absence of moesin, likely due to reduced VEGFR2 expression and activation that impair the coordinated, proliferative, and sprouting responses required for functional vessel formation.

Cell surface Ncl translocation, which is upstream of cMet activation triggered by Msn downregulation, is increased in activated endothelial cells and contributes to their angiogenic potential [10]. Cell-surface Ncl localization is also enhanced by PTN [8] or VEGFA_165_ [9] downstream of PTPRZ1, α_v_β_3_ integrin, cSrc kinase, and PI3K but not ERK1/2, similarly to the pathway identified in the present study. Intriguingly, activation of ERK1/2 occurs downstream of cell-surface Ncl expression, aligning with earlier studies reporting increased phosphorylation of ERK1/2 and Akt [37, 38], despite Ncl lacking classical signaling features. Collectively, our data suggest that plasma membrane–associated Ncl facilitates ligand-independent activation of cMet in endothelial cells. This aligns with prior research indicating that astrocyte elevated gene-1 induces Ncl-dependent tyrosine phosphorylation of cMet [39].

In addition to the interaction between Msn and α_ν_β_3_ integrin, another significant interaction, reported for the first time in the literature, is between Msn and VEGFR2, which appears to regulate VEGFR2 protein levels and localization to the cell membrane. The cell membrane and the total VEGFR2 protein levels are analogous to the Msn levels, suggesting that Msn positively regulates angiogenesis. The latter is also supported by in vitro and in vivo data from the present study, showing decreased endothelial tube formation on Matrigel, and decreased number of vessels in the growing and adult mouse brain cortices. These data are also in line with a previous study showing that Msn mediates the angiogenic effects of advanced glycation end products in vitro and in vivo [40]. Moreover, in a zebrafish moesin knockout, endothelial tubulogenesis was impaired due to the inability to maintain the apical/basal polarity of the intersegmental vessels [41].

The decreased total and cell membrane–associated levels of β_3_ integrin and VEGFR2 following Msn downregulation in endothelial cells resemble the decreased surface expression of the transferrin receptor [42] or TGF-β receptor II [43] following Msn downregulation in T cells. One explanation for such effects may be the described Msn-related regulation of the clathrin-coated vesicle trafficking [42] or its interaction with Rab11 in endosomes [44]. Activated VEGFR2 enters the Rab11-mediated recycling pathway, returning to the cell membrane and inducing endothelial permeability [45]. It is tempting to suggest that in the absence of Msn, VEGFR2 cannot be recycled to the cell membrane and is degraded in lysosomes, supported by the effect of chloroquine, which reverses the siMsn-dependent decrease in VEGFR2. Rab11-positive recycling endosomes are also involved in the recycling of integrins to the plasma membrane [46], and this may also be the mechanism through which the absence of Msn leads to decreased β_3_ integrin protein levels.

The decreased VEGFR2 expression is consistent with the reduced VEGFA_165_-induced permeability in vitro in siMsn-treated HUVEC, the increased baseline TEER in vitro, and the reduced BBB permeability observed in vivo. It also aligns with the increased ZO-1 levels in HUVEC after Msn knockdown. An inverse correlation between ZO-1 and VEGFR2 levels directly linked to vascular permeability has been reported in vitro and in vivo [47–49], and the enhanced ZO-1 levels in siMsn-treated HUVEC support the reduced basal permeability in vitro and in vivo. However, more data are needed to clarify the mechanisms by which Msn affects the levels of various proteins that regulate vascular permeability and BBB integrity. Similarly, more data are required to correlate the effects of Msn knockdown with those of the phosphorylated Msn. The latter has been linked to enhanced permeability following stimulation by various stimuli, including thrombin [50], tumor necrosis factor α [51], and advanced glycation end products [52]. Our data show that VEGFA_165_ and PTN also phosphorylate Msn at Thr558, and this may contribute to the regulation of endothelial cell permeability. On the other hand, phosphorylation of Msn at Thr558 has also been linked to compromised integrity of the vascular basement membrane [53], and this aspect is the focus of future work.

**Fig. 9:**
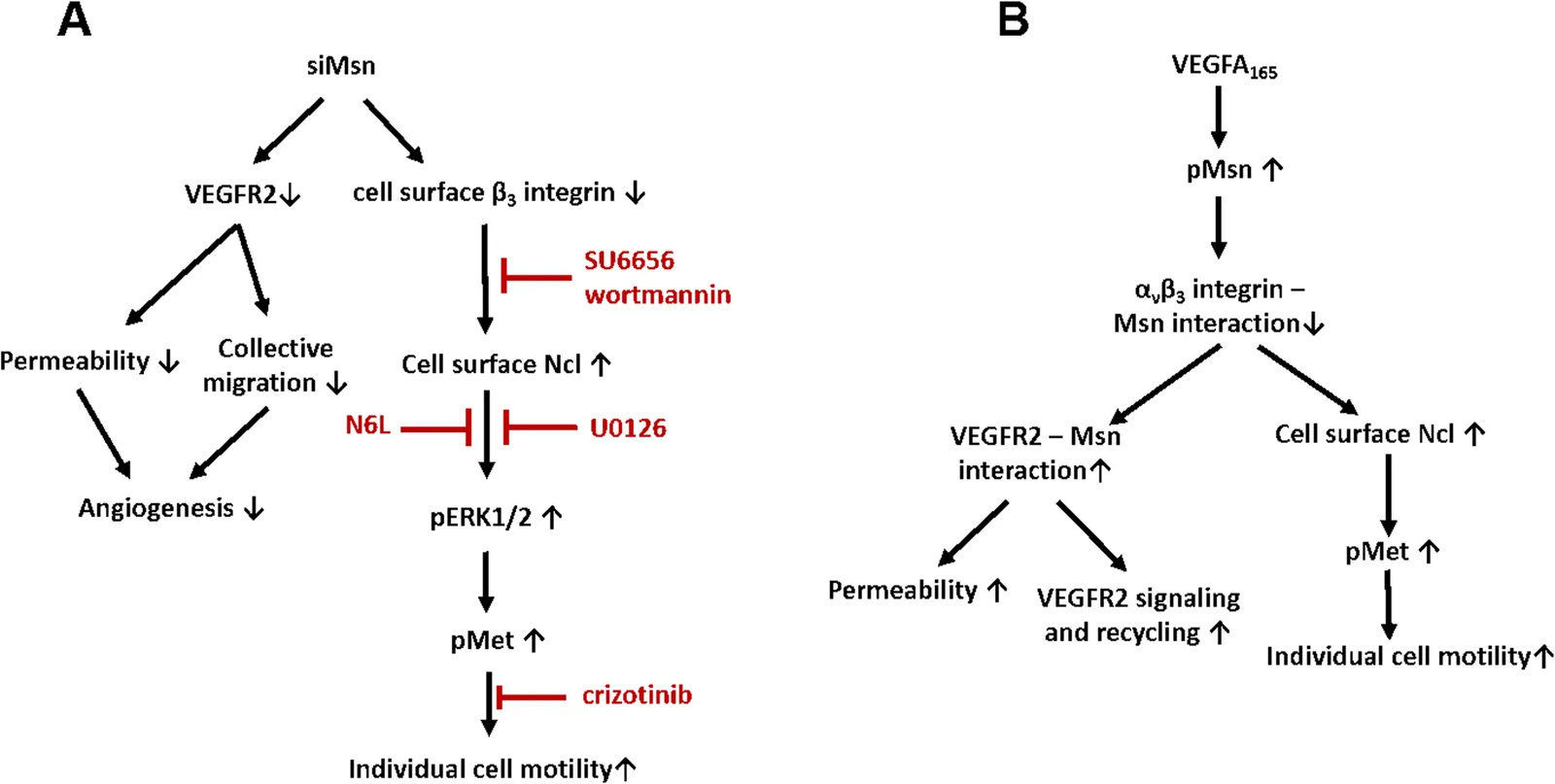
Schematic representation of pathways affected by Msn in endothelial cells. **A** Decreased Msn expression significantly reduces total and cell-surface levels of VEGFR2 and β_3_ integrin. Reduced VEGFR2 levels lead to decreased VEGFR2 tyrosine phosphorylation, impaired collective migration in scratch assays, lower permeability, and diminished angiogenesis in vivo. In contrast, decreased β_3_ integrin levels activate cSrc and PI3K, resulting in increased cell-surface Ncl, activation of ΕRΚ1/2 and cMet, and enhanced individual cell motility. **B** VEGFA_165_ enhances Msn phosphorylation at Thr558, decreases the interaction of Msn with α_ν_β_3_ integrin, and increases the interaction between VEGFR2 and Msn. This may lead to enhanced VEGFA_165_-induced endothelial cell permeability and other VEGFR2-mediated signaling relevant to angiogenesis. Together with our previous data [8, 9, 25], it is also hypothesized that the decreased interaction of Msn with α_ν_β_3_ integrin is upstream of the VEGFA_165_-induced cell surface localization of Ncl and cMet activation, which are implicated in the enhanced individual (amoeboid) cell motility induced by VEGFA_165_

Collectively, this study demonstrates that Msn interacts with the α_v_β_3_ integrin to balance c-Met and VEGFR2 activation, as illustrated in Fig. 9. These findings enhance our understanding of the pathways that coordinately regulate angiogenesis and provide insight into how potential targets can be leveraged to develop more effective anti-angiogenic strategies while minimizing resistance.

## Supplementary Information

Below is the link to the electronic supplementary material.

Supplementary Material 1

## Data Availability

The original data are provided within the article. Requests for additional information should be addressed to the corresponding author.
