# Supplementary material for: Moesin is an ανβ3 integrin-interacting protein that affects endothelial cell activation and angiogenesis through balancing c-Met and VEGFR2 activation
Source: Cell Mol Life Sci. 2026 Jul 15;83(1):371. doi: 10.1007/s00018-026-06352-5 (PMC13654922; doi:10.1007/s00018-026-06352-5)
Supplement: Supplementary file 1 [file 18_2026_6352_MOESM1_ESM.pdf]

Moesin is an $\alpha_v\beta_3$ integrin-interacting protein that affects endothelial cell activation and angiogenesis through balancing c-Met and VEGFR2 activation

Michaela-Karina Enake[1], Wanjing Lai[2,3], Sotiria Tsirmoula[1], Konstantinos Papadimatos[1], Marina Koutsioumpa[1,#], Lamprini Skondra[1], Spyros Skandalis[4], Constantinos M. Mikelis[1], Lingling Shi[2,5], Evangelia Papadimitriou[1,*]

[1]Laboratory of Molecular Pharmacology, Department of Pharmacy, University of Patras, GR26504 Patras, Greece; [2]Key Laboratory of CNS Regeneration (Ministry of Education), Guangdong Key Laboratory of Non-human Primate Research, GHM Institute of CNS Regeneration, Jinan University, Guangzhou, Guangdong 510632, China; [3]School of Biomedical Sciences, The University of Hong Kong, Hong Kong, China; [4]Laboratory of Biochemistry, Department of Chemistry, University of Patras, GR26504 Patras, Greece; [5]Department of Psychiatry, the First Affiliated Hospital of Jinan University, Guangzhou, Guangdong 510632, China

#Current address: Australis Pharmaceuticals, Inc., Simi Valley, CA, USA

[*]Author of correspondence: E. Papadimitriou, Ph.D., Laboratory of Molecular Pharmacology, Department of Pharmacy, University of Patras, GR 26504 Patras, Greece. Tel: 0030-2610-962336; e-mail: epapad@upatras.gr

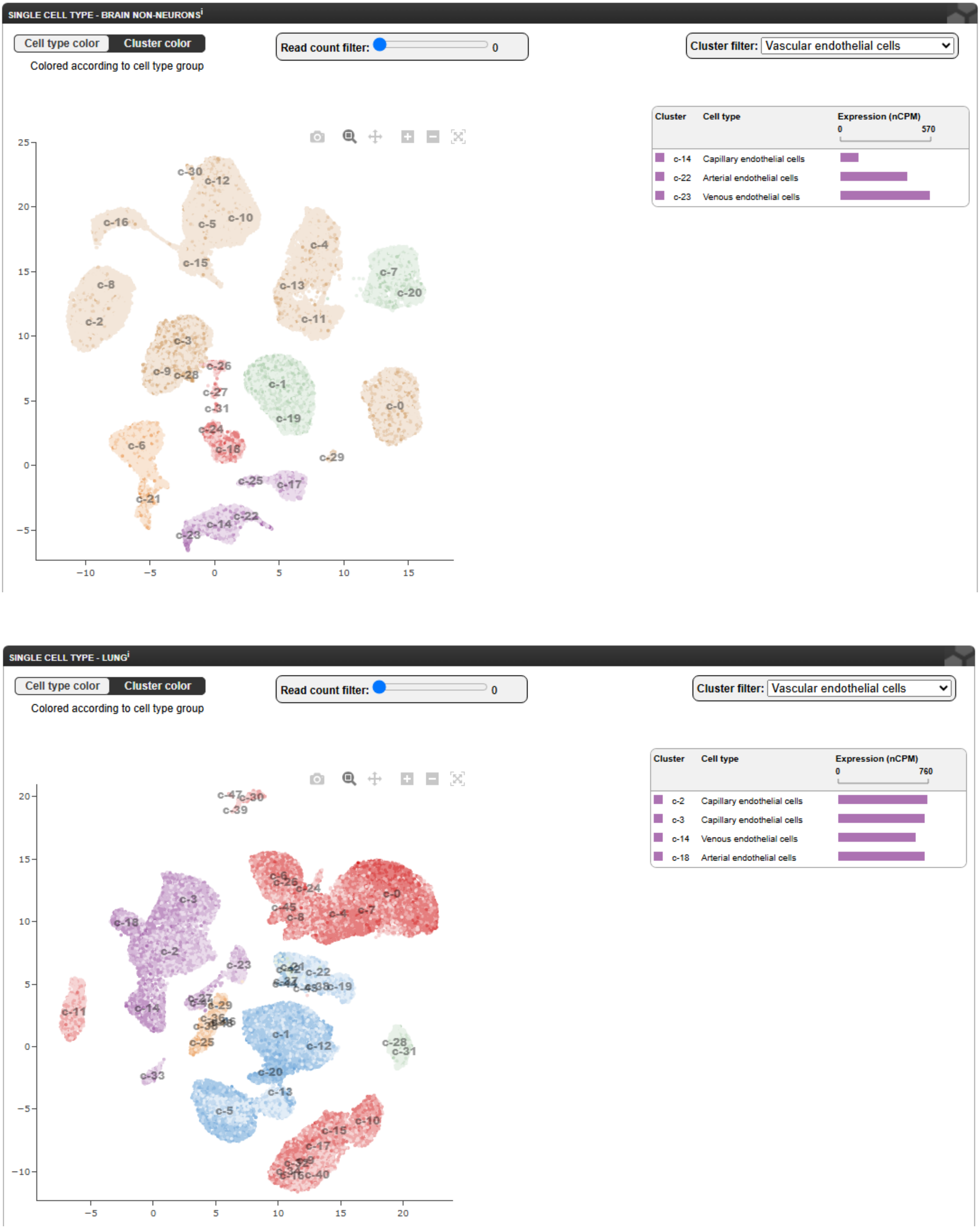

Supplementary Fig. S1. Expression of Msn in different origin endothelial cells in two tissues, brain (up) and lung (down). These data were obtained from the Human Protein Atlas, which provides additional examples from a wide range of tissues (Single cell type - MSN - The Human Protein Atlas) [28].

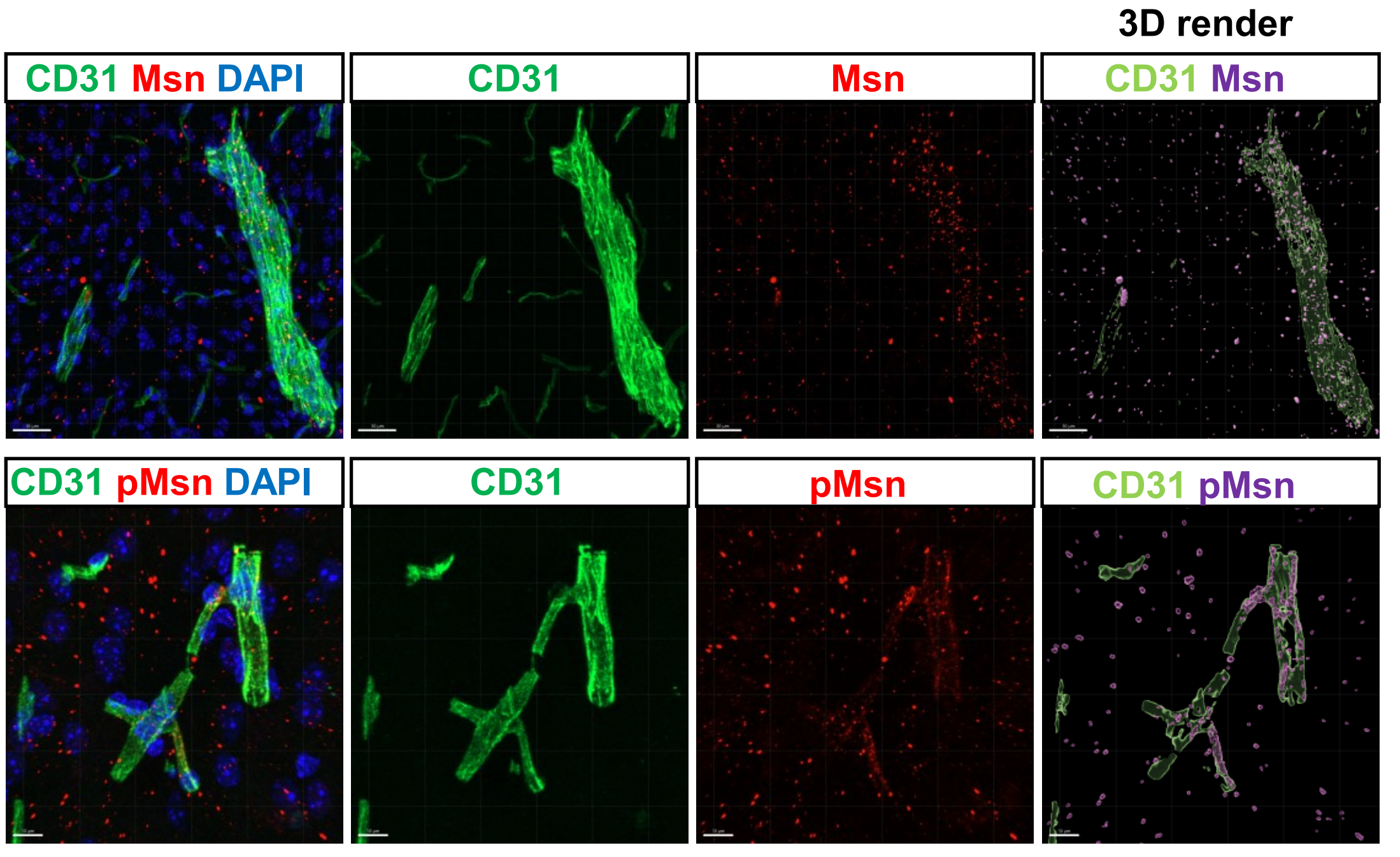

Supplementary Fig. S2A. Immunofluorescence of Msn and pMsn (red) in adult mouse cortex; CD31 marks endothelial cells (green). 3D structures rendered with Imaris. Confocal, 20×; scale bars: 30 µm (top), 10 µm (bottom).

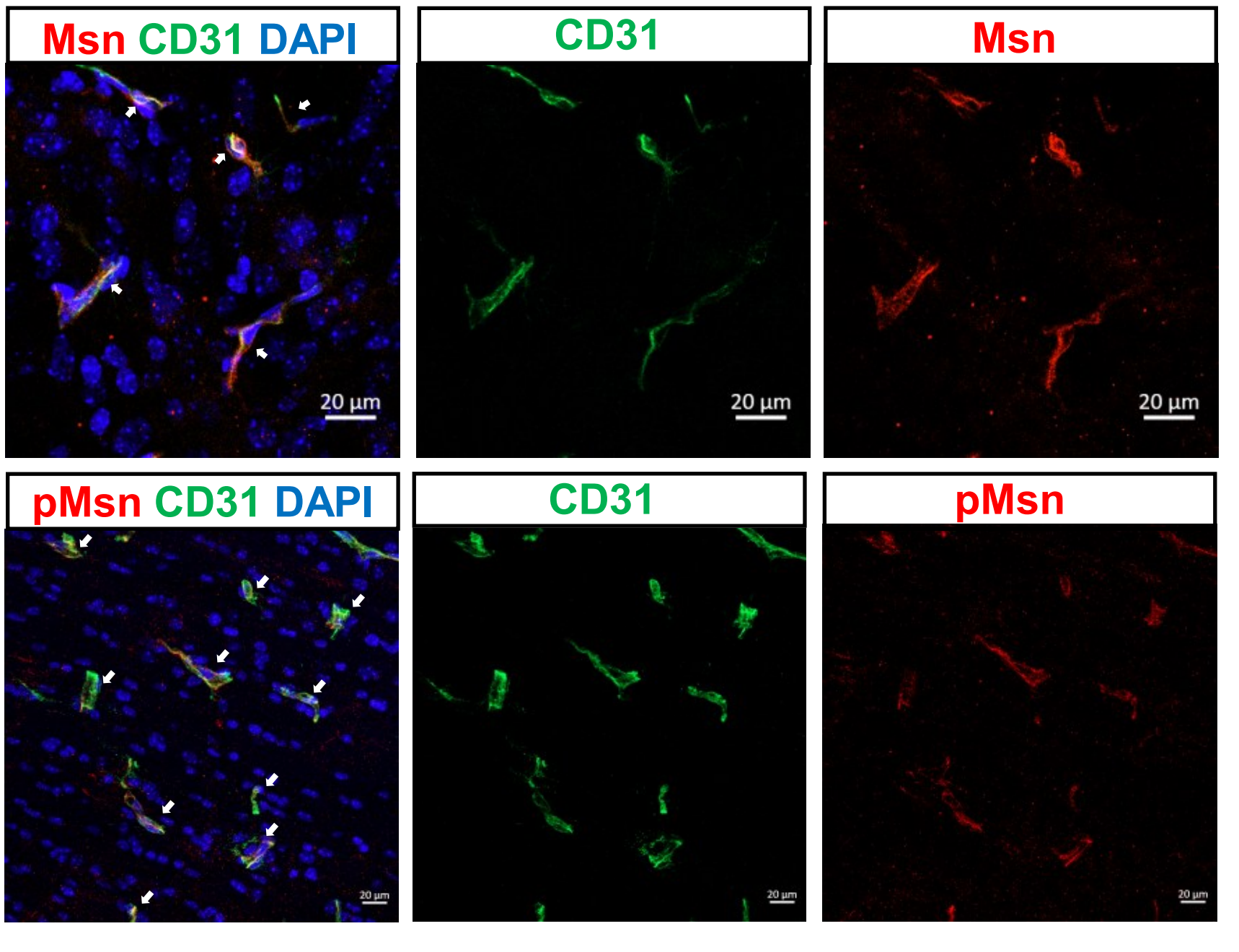

Supplementary Fig. S2B. Immunofluorescence of Msn and pMsn (red) in P7 mouse cortex; CD31 marks endothelial cells (green) — Confocal, 20×; scale bars: 20 µm. White arrows indicate co-localization.

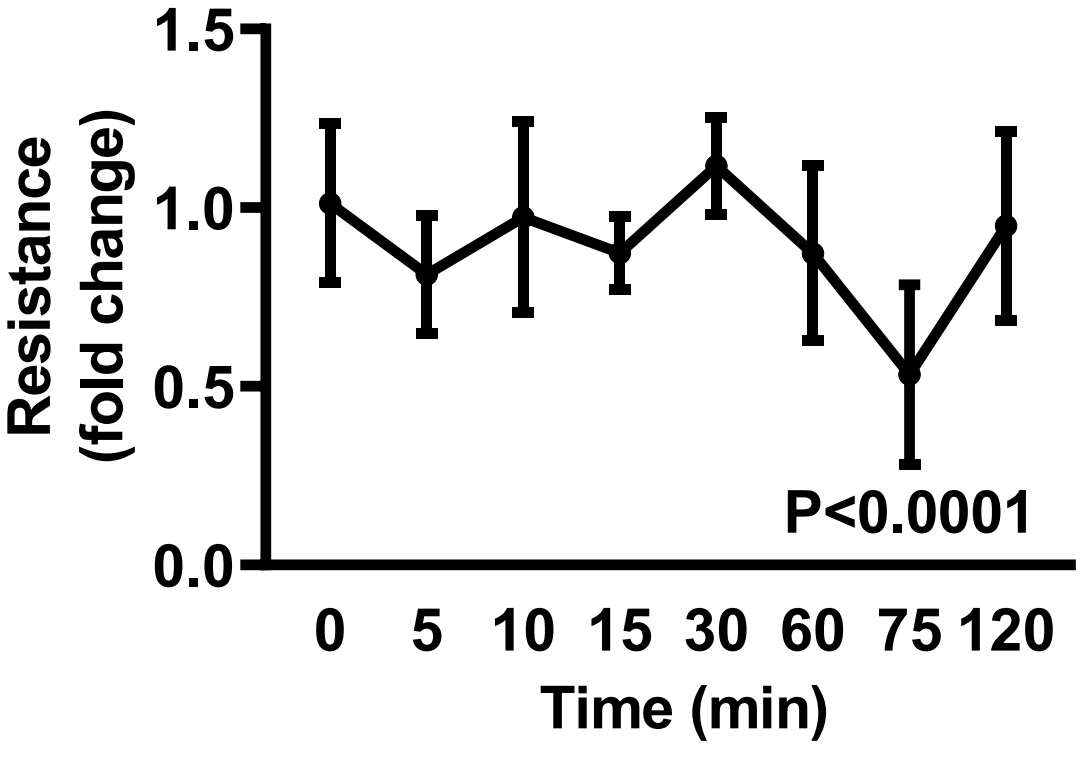

Supplementary Fig. S3. $VEGFA_{165}$ decreases TEER in HUVEC (increases permeability) 75 min following its addition to the cell culture medium. HUVEC (2 × 10^5 cells per well) were placed in 24-well microchemotaxis chambers with uncoated polycarbonate membranes featuring 0.4 µm pores. Cells were stimulated with $VEGFA_{165}$ (10 ng/mL) in serum-free medium, and permeability was assessed at different time points by measuring the TEER. Data are expressed as mean ± SD (n=4) of the fold-change compared with the untreated cells.

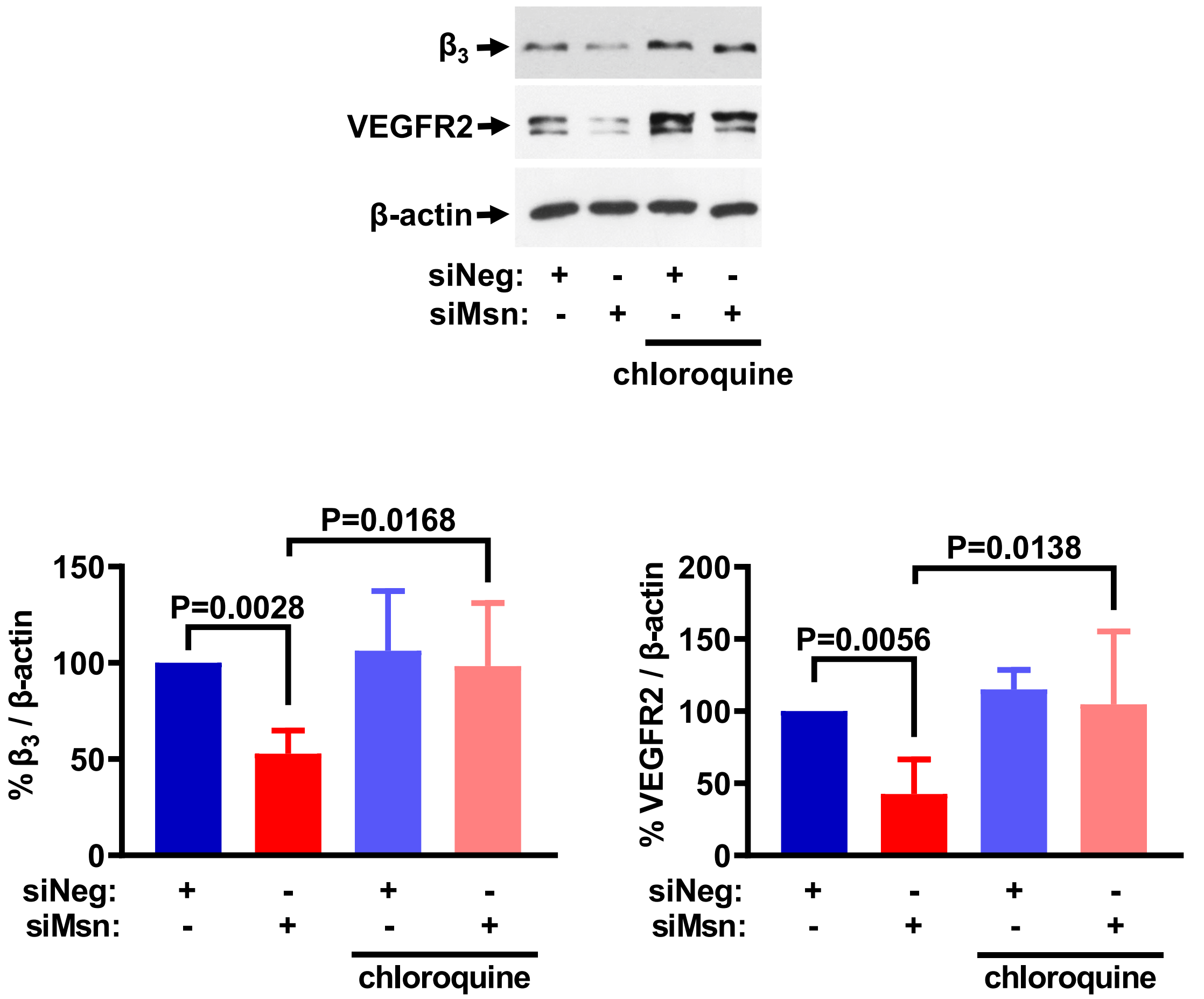

Supplementary Fig. S4. The decreased β_3 integrin and VEGFR2 protein levels following Msn downregulation are reversed by chloroquine (10 µM). (A) Total HUVEC lysates were analyzed by Western blot for β_3 integrin, VEGFR2, and β-actin (loading control). Results are expressed as mean ± SD (n=3) relative to untreated siNeg cells (set to 100%).

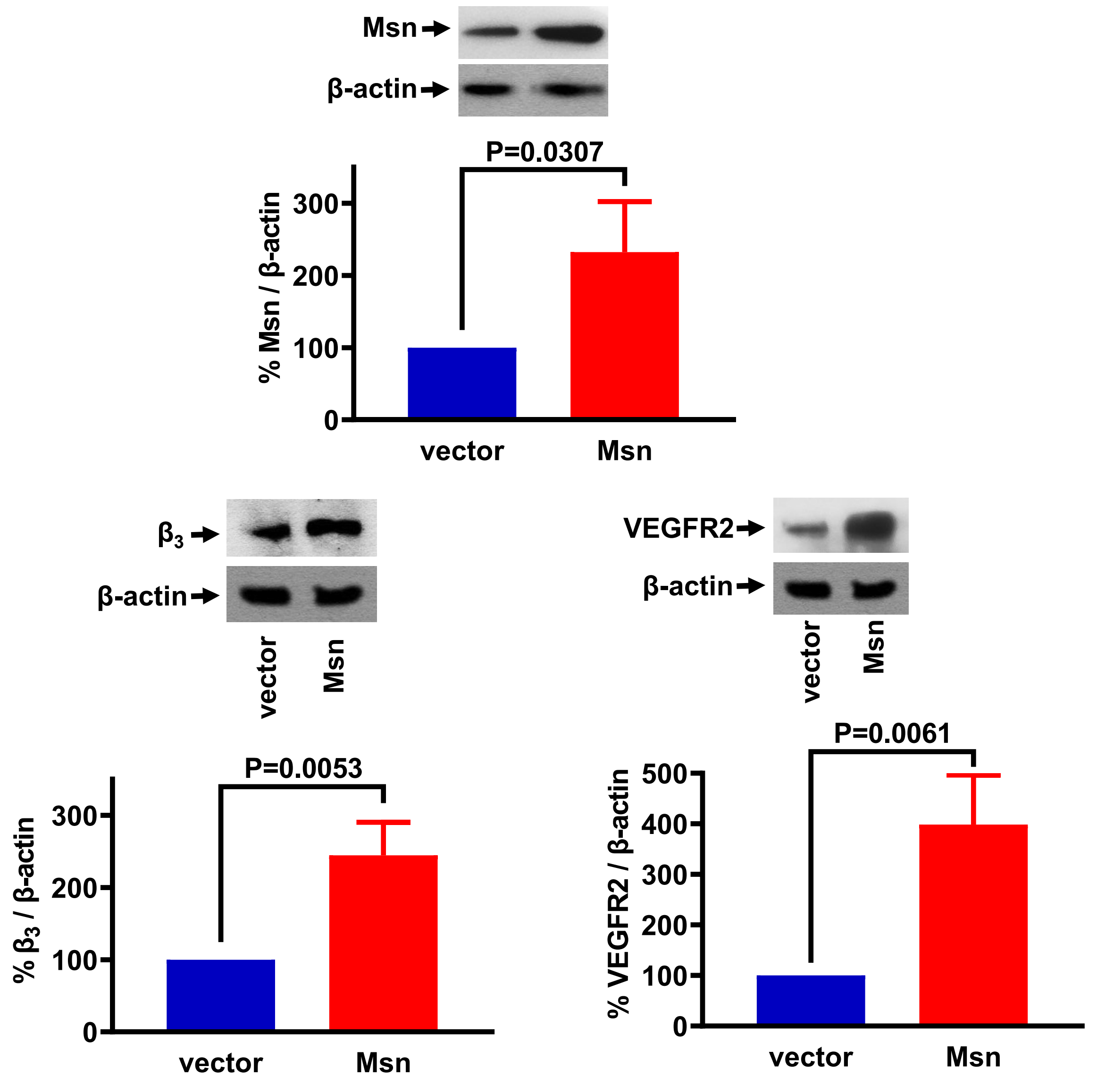

Supplementary Fig. S5. Msn overexpression leads to increased β3 integrin and VEGFR2 protein levels. Representative Western blots of total HUVEC lysates following Msn overexpression (n = 3); β-actin served as the loading control. Results are expressed as percent Msn/β-actin, β_3 integrin/β-actin or VEGFR2/β-actin ratios relative to cells transfected with the empty vector (set to 1). Vector, cells transfected with the empty plasmid vector; Msn, cells treated with the same vector carrying the human *Msn* gene.

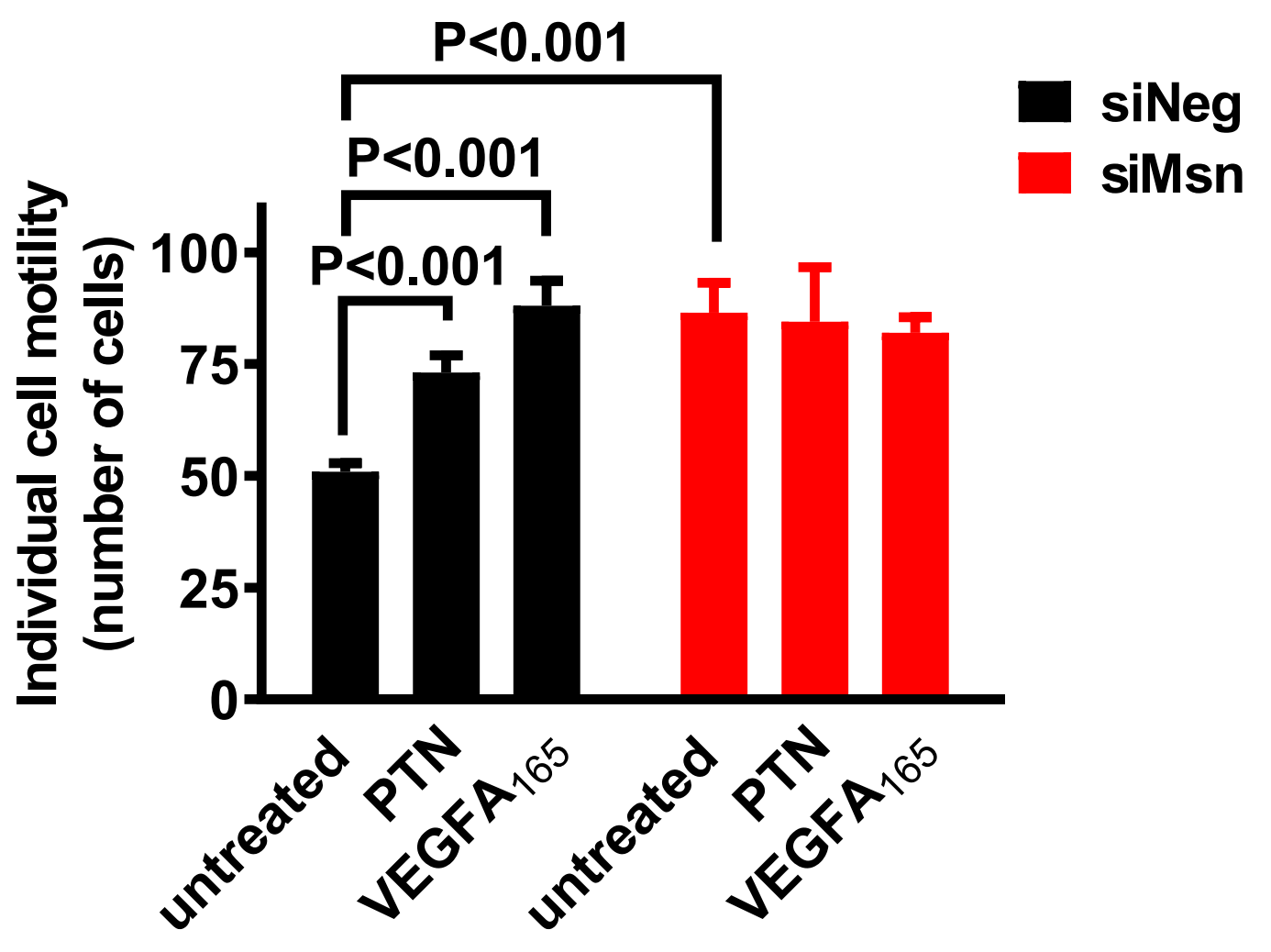

Supplementary Fig. S6. PTN or VEGFA$_{165}$ does not stimulate chemotaxis of HUVEC following down-regulated moesin expression. Following downregulation of moesin expression by siRNA, serum-starved HUVEC were stimulated with PTN (100 ng/mL) or VEGFA$_{165}$ (10 ng/mL) for 4 h in the transwell assay. Results are expressed as mean ± SD (n≥4) of the percentage of cells that migrated through the filters. siNeg, cells transfected with a negative control siRNA; siMsn, cells transfected with siRNA for moesin.

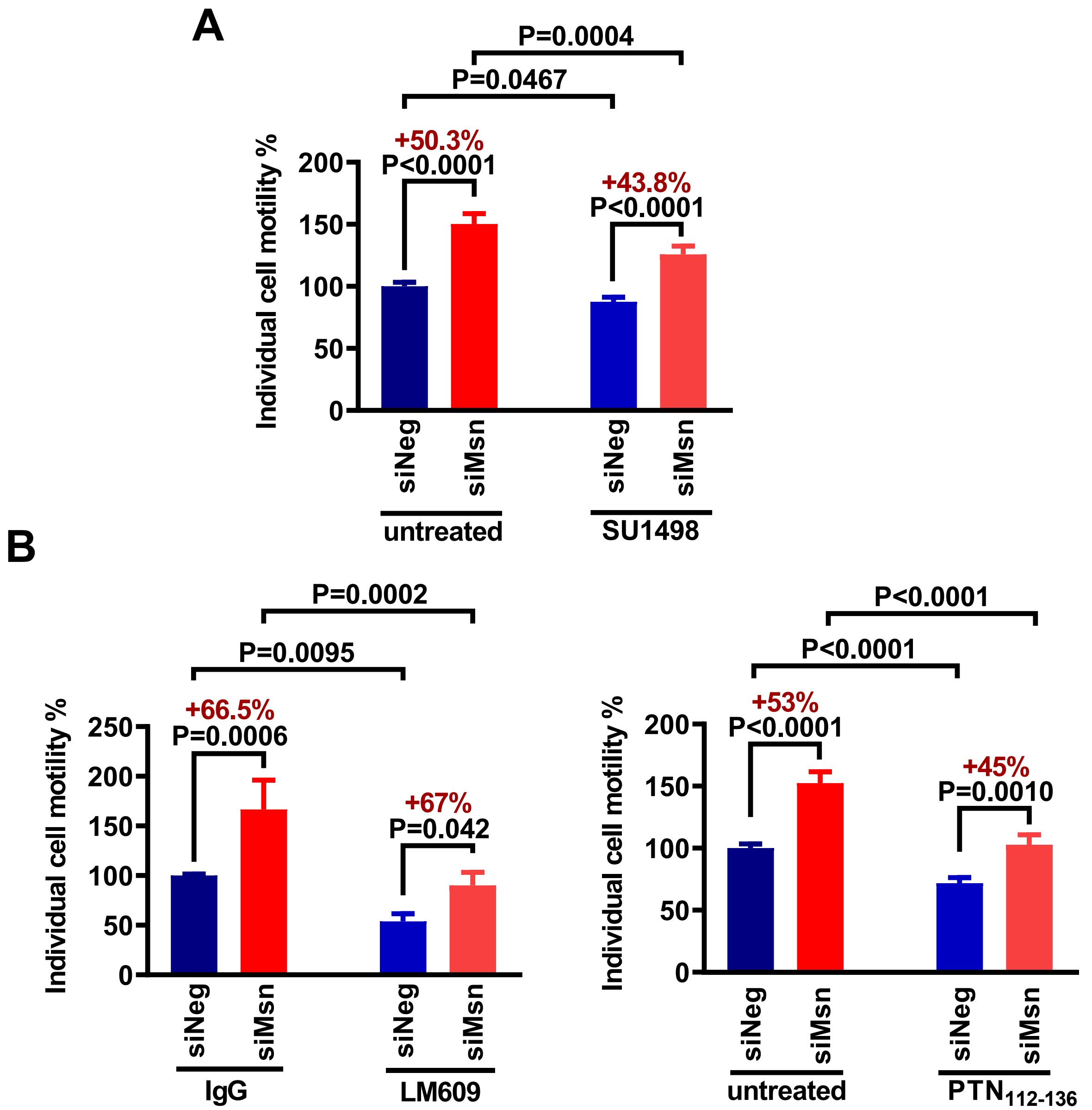

Supplementary Fig. S7. Inhibition of VEGFR2 tyrosine kinase activity (A) or $\alpha_v\beta_3$ integrin does not abolish the enhanced migration due to down-regulation of moesin expression in HUVEC. The specific VEGFR2 tyrosine kinase inhibitor SU1498 was used at 1 μM. The LM609 antibody, which binds an extracellular $\alpha_v\beta_3$ domain and inhibits ligand binding and activation (Borst et al., 2017), was used at 10 ng/mL. The PTN peptide $PTN_{112-136}$ binds to $\alpha_v\beta_3$ integrin similarly to LM609 (Mikelis et al., 2011) and was used at 100 μg/mL. Following downregulation of moesin expression by siRNA, HUVEC were treated with inhibitors or IgG (used as a negative control for LM609), and migration was measured using the transwell assay. Results are expressed as mean ± SD (n≥3) of the percentage of cells migrated through the filters compared with the untreated or the IgG-treated siNeg cells (set by default 100%). siNeg, cells transfected with a negative control siRNA; siMsn, cells transfected with siRNA for moesin.

Mikelis C, Lamprou M, Koutsioumpa M, Koutsioubas AG, Spyranti Z, Zompra AA, Spiliopoulos N, Vradis AA, Katsoris P, Spyroulias GA, Cordopatis P, Courty J, Papadimitriou E. A peptide corresponding to the C-terminal region of pleiotrophin inhibits angiogenesis in vivo and in vitro. J Cell Biochem. 2011 Jun;112(6):1532-43. doi: 10.1002/jcb.23066.

Borst AJ, James ZM, Zagotta WN, Ginsberg M, Rey FA, DiMaio F, Backovic M, Veesler D. The Therapeutic Antibody LM609 Selectively Inhibits Ligand Binding to Human αVβ3 Integrin via Steric Hindrance. Structure. 2017 Nov 7;25(11):1732-1739.e5. doi: 10.1016/j.str.2017.09.007.
